# A Lognormal Ipsative Model for Multidimensional Compositional Items

**DOI:** 10.3389/fpsyg.2021.573252

**Published:** 2021-10-12

**Authors:** Chia-Wen Chen, Wen-Chung Wang, Magdalena Mo Ching Mok, Ronny Scherer

**Affiliations:** ^1^Centre for Educational Measurement, University of Oslo, Oslo, Norway; ^2^Assessment Research Centre, The Education University of Hong Kong, Tai Po, Hong Kong, SAR China; ^3^Graduate Institute of Educational Information and Measurement, National Taichung University of Education, Taichung, Taiwan

**Keywords:** item response model, ipsative data, forced-choice items, Rasch models, compositional items

## Abstract

Compositional items – a form of forced-choice items – require respondents to allocate a fixed total number of points to a set of statements. To describe the responses to these items, the Thurstonian item response theory (IRT) model was developed. Despite its prominence, the model requires that items composed of parts of statements result in a factor loading matrix with full rank. Without this requirement, the model cannot be identified, and the latent trait estimates would be seriously biased. Besides, the estimation of the Thurstonian IRT model often results in convergence problems. To address these issues, this study developed a new version of the Thurstonian IRT model for analyzing compositional items – the lognormal ipsative model (LIM) – that would be sufficient for tests using items with all statements positively phrased and with equal factor loadings. We developed an online value test following Schwartz’s values theory using compositional items and collected response data from a sample size of *N* = 512 participants with ages from 13 to 51 years. The results showed that our LIM had an acceptable fit to the data, and that the reliabilities exceeded 0.85. A simulation study resulted in good parameter recovery, high convergence rate, and the sufficient precision of estimation in the various conditions of covariance matrices between traits, test lengths and sample sizes. Overall, our results indicate that the proposed model can overcome the problems of the Thurstonian IRT model when all statements are positively phrased and factor loadings are similar.

## Introduction

### Compositional Items in Educational and Psychological Tests

Test of non-cognitive constructs, such as personality traits (McCrae et al., 2005), attitudes (Brown and Holtzman, 1955), values (Inglehart et al., 1998), and interest (Holland, 1978), have been widely used in psychology and education and are mainly comprised of self-report items. Typical self-report items have two kinds of formats: single-stimulus and forced-choice (Vasilopoulos et al., 2006). In the single-stimulus format, respondents are asked to rate a series of items one by one according to instructions and pre-specified options. A typical options format is the Likert-type scale. In the forced-choice format, several statements are provided for each item, and respondents are asked to rank all statements (full ranking) or choose some of the statements and rank them (partial ranking) according to instructions.

Most self-report questionnaires use the single-stimulus format to assess multidimensional, non-cognitive, latent traits. For example, the NEO Personality Inventories measure five personality traits using 240 items, each of which contains five response options that range from *strongly disagree* to *strongly agree* (McCrae et al., 2005). Unfortunately, using the single-stimulus format in self-reports, tests of non-cognitive skills have several disadvantages: First, biases such as response styles (Baumgartner and Steenkamp, 2001), social desirability (Paulhus, 1991), and faking a good response (Cheung and Chan, 2002) may arise. These biases may reduce the reliability and validity of the measurement (Ganster et al., 1983). A recruitment system relying on tests might result in erroneous hiring decisions by favoring candidates who display one or more of these biases. Furthermore, the differentiation of within-person latent traits is low in the single-stimulus format (van Herk et al., 2004). For example, in a career interest test designed to provide guidance on career choices to students according to their expressed interests, a student may easily choose a constant point (e.g., the middle point of the response scale) for all items measuring different career interests. Low differentiation among different career options does not yield sufficient career choice information for the career counselor to offer any helpful advice, and further consultation may be needed as a result. Therefore, to overcome the problems of social desirability and low differentiation, implementing the forced-choice format is advisable (Nederhof, 1985; Meade, 2004; Vasilopoulos et al., 2006).

The classical scoring of forced-choice format yields ipsative scores (Brown and Maydeu-Olivares, 2011). The term “ipsative,” which means “himself” in Latin, was coined by Cattell (1944). The main feature of ipsative scores is that the sum of scores is constant (Hicks, 1970). By contrast, the single-stimulus format yields normative scores. Normative scores are mathematically independent scales; thus, a score on one scale is not the effect of a score on another scale. Normative scores enable the interpretation of scores in reference to the distribution of the population (e.g., the scores relative to the mean score for the population).

The fundamental differences between normative and ipsative scores are the referenced criterion and the explanation of the scores (Chan, 2003). For normative scores (yielded by single-stimulus format), a person’s score uses the population norm as the frame of reference (i.e., norm-referenced). The score can be explained through the comparison between individuals according to the population norm. On the contrary, ipsative scores are self-referenced in the forced-choice format. The explanation of the scores is conducted through a comparison of traits within the person. This is because, for the ipsative scale, a respondent’s score is measured relative to his/her scores on other traits.

**FIGURE 1:**
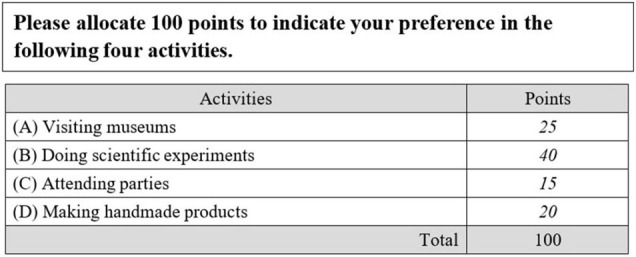
Example of compositional items.

Several types of forced-choice items have been described in the literature, such as pairwise comparison (Stark et al., 2005; Wang et al., 2017), ranking (Brown, 2016a; Wang et al., 2016), partial ranking (Hicks, 1970; Hontangas et al., 2015), and compositional items (Brown, 2016b). In pairwise comparison, respondents must choose one of two statements that best describes them or that they prefer. In ranking, respondents must rank the order of three or more statements. In partial ranking, respondents must partially order the statements rather than ordering them completely (i.e., order some rather than all of statements; Hicks, 1970). In compositional items, respondents must distribute a fixed number of points among several statements according to the extent of their latent traits (Brown, 2016b). Figure 1 shows an example of compositional items. The italicized numbers in Figure 1 are examples of responses. Apparently, compositional items result in ipsative (fixed total score of 100 in Figure 1) and continuous response data. Among the above four forced-choice formats, pairwise comparison, ranking, and partial ranking items yielded discrete response data, whereas compositional items yielded continuous response data.

An empirical example of using compositional items in the social sciences is the Organizational Culture Assessment Instrument (OCAI), through which respondents are asked to allocate 100 points to four statements in each of the six items. Each statement in an item is designed to measure a distinct dimension of organizational culture (i.e., clan, adhocracy, hierarchy, and market). Aside from its popularity in assessing organizational culture, the OCAI has been revised for assessing classroom culture (Quinn et al., 2014) and school culture (Müthing, 2013; Berkemeyer et al., 2015). To develop a good instrument, developers need a proper measurement model that is sufficient for describing the full response process (Wilson, 2005). Therefore, psychometric models with good measurement properties are needed to develop multidimensional compositional questionnaires that can provide explainable latent traits of respondents.

### Modeling Ipsative Response Data

Several models have been developed to analyze ipsative tests with categorical data, including the Thurstonian item response theory models for forced-choice items (Thurstonian IRT models; Brown and Maydeu-Olivares, 2011; Brown, 2016a), the Rasch ranking model (Wang et al., 2016), and the Rasch ipsative model (Wang et al., 2017) for dominance response and the multi-unidimensional pairwise–preference model (Stark et al., 2005) for unfolding response. Nevertheless, only Brown’s (2016b) Thurstonian model for compositional items (TMC) has been proposed for analyzing ipsative tests with continuous data.

Although the TMC (Brown, 2016b) was demonstrated to successfully recover the parameters in Brown’s simulation study, and the latent traits can be explained as the normative scores that enable between person comparisons, as a family member of Thurstonian IRT models, TMC met one problem. That is, Thurstonian IRT models require the factor loading matrix of the pairwise comparisons to have full rank (Brown, 2016a) – otherwise, convergence issues in the parameter estimation may arise (Bürkner et al., 2019). In practice, to achieve the full rank of factor loadings, one strategy is to design a combination of statements with factor loadings that are largely different from each other. Brown (2016a) proved mathematically how this strategy can solve the non-full rank problem. When the factor loadings are close to each other within ipsative items, the product of the design matrix and the matrix of factor loadings is a degenerate matrix (i.e., a matrix with columns summing to 0), because the design matrix of ipsative items is of reduced rank (Böckenholt, 2004), and the scales of the latent variables cannot be identified otherwise. Only when factor loadings are considerably different from each other in ipsative items, the product of the design matrix and the matrix of factor loadings has full rank, and then the factor covariance matrix can be identifiable. To implement this strategy empirically, item contents should be written carefully for manipulating the factor loadings. For example, an item can be composed of a negatively keyed statement (negative loading) and a positive keyed statement (positive loading). Another example is to use a number of “distractor” items with zero loadings on traits to be measured when the negative keyed statements are not desired. Although there are psychometric tests with all positive statements (matched on social desirability) that produce normative factor scores using Thurstonian IRT successfully, for instance, OPQ32r assessing millions of candidates per year since 2009 (e.g., Joubert et al., 2015), forced-choice tests with all positive loadings may not necessarily satisfy the full-rank criterion of the loading matrix.

One way for test developers to overcome the non-full rank of loading matrix problem in Thurstonian IRT is to increase the number of latent traits measured in the test (Bürkner et al., 2019). However, when researchers do not collect data by themselves, they have to rely on the assessment data and the framework underlying it – in such scenarios, it is often impossible to increase the number of traits in the test. For example, the Programme for International Student Assessment (PISA; OECD, 2014) measured students’ mathematics intentions by forced-choice items with only three traits in 2012.

Including the negative keyed statements in forced-choice items is another way to satisfy the full-rank requirement. However, it is still a risk to meet the identification problem when the number of dimensions is low. Bürkner et al. (2019) found that only half of the iterations in simulations of Thurstonian IRT models converged when tests included the unequally keyed statements in the design and measured only five traits. The failure of convergence is unacceptable in practical testing, especially in the time-consuming test construction procedure and in high-stakes situations. On the other hand, the unequally keyed statement design would undermine the purpose of forced-choice tests that are meant to reduce the social desirability bias that occurs when the negative keyed statement can be easily identified.

In contrast, the Rasch ipsative model (RIM; Wang et al., 2017) does not suffer from the problem of bias estimation when using equally keyed statements or the problem of convergence. RIM essentially assumes all statements have the same key over the test and always converge regardless the true values of parameters. Mathematically, the RIM can be considered as a special case of Thurstonian IRT models with all factor loadings fixed to one and ipsative constraints in latent variables. The limitation of RIM is that the normative scores were no longer produced, and the scores in RIM can only be interpreted in the ipsative way. Additional benefit of RIM is that it satisfies the good measurement property of specific objectivity (Rasch, 1977). Wang et al. (2017) have demonstrated that the sample-free and test-free properties (i.e., specific objectivity) cannot be satisfied by the models for force-choice items in the Thurstonian framework. The RIM works for only the discrete ipsative data. A measurement model for continuous ipsative data (i.e., compositional data) overcoming the non-full rank loadings problem of TMC is a knowledge gap in the literature.

### The Present Study

In the present study, we mimic the works of RIM study but for the compositional data. More specifically, this study is aimed at developing a measurement model for multidimensional compositional items as an alternative version of Thurstonian IRT models. The new model is mathematically nested in the TMC (Brown, 2016b), but has fundamentally different measurement properties. The new model resolves the non-full rank loading matrix problems of the TMC. We show that the new model has good properties with regards to specific objectivity, parameter recovery in equally keyed statements situations (i.e., the factor loadings all equal to one), and model convergence in the analysis of both the real and the simulated data. In this study, the analysis of the real data illustrates the interpretation of item parameters and latent traits, and the simulation study demonstrates the model performance with respect to parameter recovery and model convergence. For simplicity, all the compositional items mentioned in this article indicate multidimensional compositional items rather than unidimensional compositional items.

The remainder of this article is organized as follows: We first discuss the theoretical background and introduce the compositional analysis, along with Brown’s (2016b) TMC. Then, we explain the proposed compositional model and its specific objectivity. Comparisons are made between the proposed model and the TMC. The methods used to evaluate the model fit of the compositional data and used in the analysis of the empirical data are presented. We further present the empirical study to illustrate the application and implications of the model in practice, using real data. The subsequent section describes the simulation studies that were conducted to demonstrate the parameter recovery of the proposed model compared to the TMC. Finally, we discuss our findings and elaborate on their practical implications.

## Background

### Compositional Data Analysis

Compositional data are defined as a vector of *D* positive continuous numbers **X** = [*X*_1_, …, *X*_*D*_], where the sum of the components is a constant *C* (Aitchison, 1986). The practice of requiring components to sum to a constant, especially the components measured in percentages (i.e., summing to 100%), is widespread in the geosciences, geology, and other disciplines (e.g., Stephens and Diesing, 2015).

To parametrically model compositional data, Aitchison (1986) introduced the additive log ratio transformation to simply transfer the compositional data to a normal distribution. The additive log ratio is used for transferring the *D* dimensional components of **X** = [*X*_1_, …, *X*_*D*_] to the logarithm of the remaining *D*–1 components by dividing each of them by the *D*th reference component before taking the log. The process is expressed as follows:

(1) Additivelogratio(X,1…,X)D=[lnX1XD,⋯,lnXD-1XD]

In psychological measurements, Brown (2016b) also used the Thurstonian IRT model framework to function the additive log ratio transformed data. In this research, we propose the item response theory (IRT) model for multidimensional compositional items that fall under the additive log ratio framework as well.

### Thurstonian Model for Compositional Items

As mentioned, the TMC (Brown, 2016b) is the only IRT model that has been developed for compositional items. To illustrate Brown’s (2016b) model, let a multidimensional compositional item have *D* statements. The *d*th statement (*d* = 1, …, *D*) measures the latent trait *θ_*d*_*. Let *X*_1_, …, *X*_*D*_ denote the responses to statements 1, …, *D*, respectively. For simplicity, we do not index the persons and items unless necessary. With one statement (e.g., statement *D* arbitrarily) in the item taken as a reference, the log ratio of the responses *X*_*k*_ (*k* = 1, …, *D* − 1) to response *X*_*D*_ can be written as follows:

(2) $$Y_{k\,D}\equiv \log\left(X_{k}/X_{D}\right)=\log\left(X_{k}\right)-\log\left(X_{D}\right)$$

where *Y*_*kD*_ is the additive log ratio transformation and is assumed to follow a multivariate normal distribution (Aitchison and Shen, 1980). Brown (2016b) proposed the accounting for *Y*_*kD*_ as follows:

(3) $$Y_{k\,D}=\log\left(X_{k}\right)-\log\left(X_{D}\right)=\delta_{k\,\_\,D}+\beta_{k}\,\theta_{k}-\beta_{D}\,\theta_{D}+\varepsilon_{k\,\_\,D}$$

where δ*_*k*_*___*_*D*_* is the utility (location) of statement *k* relative to the reference statement *D*; β*_*k*_* and β*_*D*_* are the discrimination (slope) of statement *k* and statement *D*, respectively; θ*_*k*_* and θ*_*D*_* are the latent traits in dimension *k* and *D*, respectively; and ε*_*k*_*___*_*D*_* is the error term.

Essential to the analysis of ipsative data is that only within-person comparisons, rather than between-persons comparisons, can be concluded from the scores (Hicks, 1970). Brown (2016b) claimed that the TMC could overcome this limitation and pointed out the following feature of her model: “Thus, ipsative data do not arise, and interpersonal comparisons can be made.” To confirm this feature, she worked on obtaining a good parameter recovery of the models in a simulation study. Even if the controversy of whether the ipsative nature was maintained is overlooked, Brown’s study using the TMC aimed to make comparisons between measures. The comparison of scores between individuals is attractive to the practitioners using non-cognitive tests with forced-choice items for the purpose of evaluating work performance and career development (Joubert et al., 2015; Merk et al., 2017; Guenole et al., 2018).

The models in the Thurstonian IRT model framework (Brown, 2016b) cannot satisfy the property of specific objectivity (Wang et al., 2017). Theoretically, a measurement model without specific objectivity fundamentally does not allow meaningful comparisons to be made between measures. RIMs do enable such comparisons to be made, however, it is not a sufficient reason to prefer the use of RIMs over Thurstonian models. There are dozens of very important measurement models out there that do not embrace specific objectivity. It does not mean they have not considered specific objectivity. Instead, they have emphasized reasons for not taking it, such as Brown’s (2016b) TMC that has a measurement goal of producing ipsative data as normative scores for making individual comparison. Therefore, when doing the ipsative response data analysis we have a dilemma – do we want specific objectivity, or do we want normative data? If the purpose of measurement is obtaining within-person preferences with good control of item properties, then the specific objective model (i.e., RIM) might be preferred. If the purpose is establishing normative scores for people on traits, then the specific-objective model cannot deliver, and one needs to use Thurstonian IRT models.

## Developing the Lognormal Ipsative Model (LIM) for Compositional Items

The RIM is only used for analyzing discrete ipsative response data. In this section, a new model for the analysis of compositional items under the RIM framework is introduced. The model, that is, the lognormal ipsative model (LIM), the parameter estimation method, and the calculation of the approximate standard error, and the Fisher information function are all described. Point estimates (ipsative explanation) are then explained. Subsequently, we compare the new model and the Thurstonian model and present a method for evaluating the fit of the new model.

### The Lognormal Ipsative Model

According to the additive log ratio transformation (Aitchison, 1982), the compositional response ***X*** = [*X*_1_, …, *X*_*D*_] with *D* elements can be transferred to $Y=\left[\log\frac{X_{1}}{X_{D}},\cdots ,\log\frac{X_{D-1}}{X_{D}}\right]$ with *D* − 1 elements, where the reference response *X*_*D*_ is arbitrarily selected from *X*_1_, …, *X*_*D*_. We can express *Y* as the following log ratio function as Eq. 2 where *k* (*k* = 1, …, *D*–1) indexes any of the dimensions other than the reference dimension *D*. The log ratio **Y** works on the assumption of a multivariate normal distribution. Conceptually, in the context of multidimensional compositional items, the responses *X*_*k*_ and *X*_*D*_ should be decided by the three effects of a person’s latent trait θ in the corresponding dimension, statement utility δ, and random error ε. For simplicity, we do not index the persons and items until necessary. Following the argument above, the LIM decomposes log(*X*) as follows:

(4) $$\log\left(X_{k}\right)=\theta_{k}+\delta_{k}+\varepsilon_{k},\log\left(X_{D}\right)=\theta_{D}+\delta_{D}+\varepsilon_{D}$$

Thus,

(5) $$\begin{array}{c} Y_{k\,D}=\log\left(X_{k}\right)-\log\left(X_{D}\right)\\ =\left(\theta_{k}+\delta_{k}+\varepsilon_{k}\right)-\left(\theta_{D}+\delta_{D}+\varepsilon_{D}\right)\\ =\left(\theta_{k}+\delta_{k}\right)-\left(\theta_{D}+\delta_{D}\right)+\varepsilon_{k\,D}, \end{array}$$

where θ*_*k*_* and θ*_*D*_* are a person’s latent traits on dimensions *k* and *D*, respectively; δ*_*k*_* and δ*_*D*_* are the utilities of statements *k* and *D*, respectively; and ε*_*kD*_* is the error term following a normal distribution with a mean of zero and variance of $\sigma_{\varepsilon }^{2}$.

**FIGURE 2:**
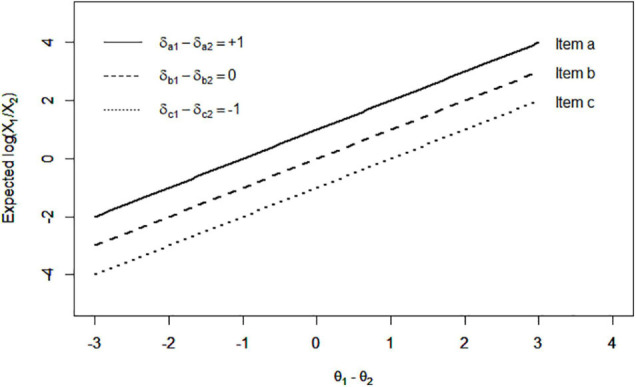
Expected log ratio of X_1_ to X_2_ across different levels of θ_1_–θ_2_ and δ_1_–δ_2_ in the LIM.

To illustrate this model simply, let us take the item composed of statements 1 and 2 (i.e., *D* = 2) as an example. Figure 2 presents the monotonically increasing response function of the LIM. The horizontal axis is the difference of θ_1_ and θ_2_, which represents a person’s pattern of latent traits. Specifically, a larger θ_1_–θ_2_ leads to a higher expected value of the log ratio of X_1_ to X_2_ (the positive slope in Figure 2). As a result, a respondent having larger θ_1_–θ_2_ is expected to give a higher value to X_1_ than to X_2_ across the different δ levels. Thus, the between-persons comparison is enabled on the pattern of θs. Moreover, a larger δ_1_–δ_2_ (the higher line in Figure 2) leads to a higher expected value of log(X_1_/X_2_), which is also monotonically increasing. The item with a positive δ_1_–δ_2_ enables persons to give a higher value to X_1_ than to X_2_ across θ levels.

The LIM yields the unique utility for each individual statement that is different from the Thurstonian model yielding utilities (location parameters) for pairs of statements. As can be seen in Eq. 5, δ*_*k*_* and δ*_*D*_* are obtained as the utility for the statements *k* and *D* regardless which compositional items. The amount of unique utility for either statement *k* or statement *D* is modeled so the statements are allowed to be used repeatedly among items. The repetition of using common statements shared among items does not violate the assumption of local independence in the parameter estimation.

For the model identification, the following two constraints are necessary. The first one is $\sum_{d=1}^{D}\theta_{d}=0$ for every person, which also considers the ipsative nature that $\sum_{d=1}^{D}X_{d}=C$ for every person. The second one is $\sum_{i=1}^{I}\delta_{i\,d}=0$ for every dimension *d* (*d* = 1, …, *D*), where δ stands for the statement utility, *i* indexes the items, and *I* is the total number of items. In other words, the sum of the statement utilities across items should be fixed to zero for each dimension.

The LIM has the property of specific objectivity. The sample-free and item-free properties of specific objectivity for compositional data analysis can be found in Appendix A. Suppose persons *n* and *m* respond to the identical item *i* with *D* statement. For them, the expected log ratio of *Y*_*kD*_ following from Eq. 5 is as follows:

(6) $$\begin{array}{l} E(Y_{ni(kD)})=({\text{θ}}_{n(k)}+{\text{δ}}_{i(k)})-({\text{θ}}_{n(D)}+{\text{δ}}_{i(D)})\text{ and}\\ \;E(Y_{mi(kD)})=({\text{θ}}_{m(k)}+{\text{δ}}_{i(k)})-({\text{θ}}_{m(D)}+{\text{δ}}_{i(D)}) \end{array}$$

The test-free measurement is demonstrated by comparing two persons such that

(7) $$\begin{array}{l} \log\left[\frac{X_{ni(k)}/X_{ni(D)}}{X_{mi(k)}/X_{mi(D)}}\right]=E(Y_{ni(kD)})-E(Y_{mi(kD)})\\ =({\text{θ}}_{n(k)}-{\text{θ}}_{n(D)})-({\text{θ}}_{m(k)}-{\text{θ}}_{m(D)}) \end{array}$$

This expression is independent of the item parameters δ*_*i*_*_(_*_*k*_*_)_ and δ*_*i*_*_(_*_*D*_*_)_, which is the requirement of test-free property for compositional data. The measurement satisfies the test-free property. Similarly, to demonstrate the sample-free of the LIM, when person *n* responds to two items, *i* and *j*, the expected log ratio follows from Eq. 5:

(8) $$\begin{array}{l} E(Y_{ni(kD)})=({\text{θ}}_{n(k)}+{\text{δ}}_{i(k)})-({\text{θ}}_{n(D)}+{\text{δ}}_{i(D)})\text{ and}\\ \;E(Y_{nj(kD)})=({\text{θ}}_{n(k)}+{\text{δ}}_{j(k)})-({\text{θ}}_{n(D)}+{\text{δ}}_{j(D)}) \end{array}$$

Then,

(9) $$\begin{array}{l} \log\left[\frac{X_{ni(k)}/X_{ni(D)}}{X_{nj(k)}/X_{nj(D)}}\right]=E(Y_{ni(kD)})-E(Y_{nj(kD)})\\ =({\text{δ}}_{i(k)}-{\text{δ}}_{i(D)})-({\text{δ}}_{j(k)}-{\text{δ}}_{j(D)}) \end{array}$$

This expression is independent of the person parameters θ*_*n*_*_(_*_*k*_*_)_ and θ*_*n*_*_(_*_*D*_*_)_, which is the requirement of sample-free for compositional data. Therefore, the LIM is a sample-free model and satisfies the property of specific objectivity. Conceptually, specific objectivity in the LIM implies that the comparison between persons’ patterns of the latent traits (i.e., comparison between person *n*’s profile θ*_*n*_*_(_*_*k*_*_)_–θ*_*n*_*_(_*_*l*_*_)_ and person *m*’s profile θ*_*m*_*_(_*_*k*_*_)_–θ*_*m*_*_(_*_*l*_*_)_) is under a scale with the measurement property of test-free (see Appendix A), and that the comparison between statement utilities (i.e., comparison between statement *k*’s utility δ_(_*_*k*_*_)_ and statement *l*’s utility δ_(_*_*l*_*_)_) remains stable even when different persons take the test.

### Parameter Estimation

This study used the Bayesian approach of the Markov chain Monte Carlo (MCMC) algorithm for parameter estimation in the analysis of the empirical data. The method of posterior predictive model checking (PPMC) was adopted in the evaluation of model–data fit, and it was administered effectively in the MCMC iterations (Levy et al., 2009). The existing model – data fit for IRT model such as Yen’s Q1 (Yen, 1981), S-χ^2^ (Orlando and Thissen, 2000), and M_2_ statistic (Maydeu-Olivares and Joe, 2006) are not adopted in this study because they are used to examine model-misspecification for dichotomous or polytomous items and not to examine model–data fit with continuous response data.

The MCMC estimation utilized the Bayesian framework and was sampled from the joint posterior distribution of the parameters. To make it applicable to the new model, the joint posterior distribution, given the whole data set **X**, is written as follows:

(10) P⁢(θ,δ|X)∝L⁢(X|θ,δ)⁢P⁢(θ|μ,Σ)⁢P⁢(μ|Σ)⁢P⁢(Σ)⁢P⁢(δ)

where **θ***_*n*_* is the person’s latent traits vector with *D*–1 elements following a multivariate normal distribution with mean **μ** and variance **Σ**, and **δ** is the utility vector of statements. *L*(*X*|θ,δ) is the likelihood function based on the fitting model, given the assumptions of local independence and independence of responses between persons. The *P*(θ|μ,**Σ**) is the conditional probability for person’s latent traits θ, and *P*(**μ**| **Σ**), *P*(**Σ**), and P(**δ**) are the prior distributions for **μ**, **Σ**, and **δ**.

For the LIM, the likelihood function is

(11) $$L\,\left(\text{X}|\theta ,\delta \right)=\prod_{n=1}^{N}\prod_{i=1}^{I}\prod_{d=1}^{D-1}\frac{1}{\sqrt{2\,\text{π}\,\sigma_{\varepsilon }^{2}}}\exp\left\{-\frac{{\left[\ln\left({\text{X}}_{d}\,/\,{\text{X}}_{D}\right)-\left(\theta_{n\,d}+\delta_{i\,d}\right)+\left(\theta_{n\,D}+\delta_{i\,D}\right)\right]}^{2}}{2\,\sigma_{\varepsilon }^{2}}\right\}$$

Using the Metropolis-Hastings algorithm with the Gibbs sampling procedure allows for the sampling and the obtaining of the full conditional distributions of parameters **θ** and **δ**. In the estimation of **θ**, only *D*–1 elements (denoted as **θ**_–_*_*D*_* for convenience) need to be estimated because of the ipsative constraint. The prior distributions were set the same with the RIM study by Wang et al. (2017). The prior distribution of **δ** follows standard normal distribution. The prior distribution of **θ_–_***_*D*_* follows a multivariate normal distribution with a mean **μ** = [μ_1_, …, μ_*D*__–__1_]^*T*^ and a covariance matrix **Σ**. The hyperprior distribution of elements in **μ** is *N*(0, 1), and the hyperprior distribution of **Σ** is the inverse Wishart distribution [**R**, K], with **R** = **I** and the hyperparameter *K* = *D*−1. The prior for the error variance $\sigma_{\varepsilon }^{2}$ is an inverse gamma distribution with shape and rate parameters both equal to one.

A popular use of the expectation-maximization algorithm (EM algorithm) for the IRT model (Muthén and Muthén, 1998–2012) was not adopted because as the number of dimensions in the model increases, the EM algorithm becomes more difficult computationally in the application. Unfortunately, non-cognitive tests usually involve multidimensionality. Furthermore, this approach does not incorporate the uncertainty of item estimates into the estimation of person parameters (Patz and Junker, 1999). The reason is that the EM algorithm is first used to estimate the item parameters by the marginal distribution of persons’ latent traits. Then, the person estimates are obtained by fixing the item estimates in the iterations of the algorithm. The standard error of the item estimates does not take into account the person estimates. Conversely, using MCMC to jointly estimate the person and item parameters does not suffer from this problem.

### Approximate Standard Error and Fisher Information

In Bayesian estimation, the standard error of estimates can be obtained by calculating the variance of posterior distribution. In maximum likelihood estimation the diagonal elements of the square root of the inverse Fisher information represent the approximate standard error of the multidimensional estimates (Fisher, 1922). This section describes the calculation of the approximate standard error and the Fisher information functions for the LIM.

Given the response vector **X**, person’s θ, and statement δ, the log-likelihood of a person answering *I* items can be expressed as follows:

(12) $$\ln L\left(\theta ;X\right)=-\frac{1}{2}\sum_{i}^{I}\sum_{d=1}^{D-1}[\ln\sigma^{2}+\ln\left(2\pi \right)+{\left(y_{i\,d\,\_\,D}-{\hat{y}}_{i\,d\,\_\,D}\right)}^{2}/\sigma^{2}]$$

where *D* is the reference dimension; *d* is any dimension other than *D*; *y*_*id*_*D*_ = *ln*⁡(*X*_*id*_/*X*_*iD*_) is the log ratio of response *X*_*id*_ to response *X*_*iD*_ in item *i*; ${\hat{y}}_{i\,d\,\_\,D}=\left(\theta_{d}+\delta_{i\,d}\right)-\left(\theta_{D}+\delta_{i\,D}\right)$; θ*_*d*_* and θ*_*D*_* are latent traits in dimensions *d* and *D*, respectively; δ*_*id*_* and δ*_*iD*_* are the utilities of *d* and *D* statements in item *i*, respectively; and σ^2^ is the residual variance. The second derivative of the log-likelihood for the *k*th latent trait θ*_*k*_* is formed as $\frac{\partial^{2}\ln L}{\partial \theta_{k}^{2}}=\frac{-I}{\sigma^{2}}$, which is a function that only involves the residual variance σ^2^ and the current test length *I*. The Fisher information matrix of the LIM for each item is written as

(13) $$\text{I}\,\left(\theta \right)=-E\,\left[\frac{\partial^{2}\ln L}{\partial \theta \,\partial \theta^{\prime }}\right]=\left[\begin{array}{cc} 1\,/\,\sigma^{2} & 0\\ 0 & 1\,/\,\sigma^{2} \end{array}\right]$$

Note that the 2 × 2 matrix in *D*-dimensional model represents the reference dimension *D* and any dimension *d* other than *D* for the expression of a multidimensional model rather than for a model only including two dimensions. After administering a set of *S* items, the test information matrix takes the following formml:

(14) $${\text{I}}_{S}\,\left(\theta \right)=\sum_{i\in S}{\text{I}}_{i}\,\left(\theta \right)=\left[\begin{array}{cc} I\,/\,\sigma^{2} & 0\\ 0 & I\,/\,\sigma^{2} \end{array}\right]$$

where *I* is the test length, implying that a longer test length results in higher test information. This expression corresponds to the assumption of linear regression that the residual variance is independent of the predictors (which can be latent or observed) and is only related to the number of observations.

The approximate standard error of the estimate is the diagonal elements of the square root of the inverse Fisher information:

(15) $${\hat{\sigma }}_{\theta_{k}}={\left\{-E\,\left[\partial^{2}\ln L\,/\,\partial \theta_{k}^{2}\right]\right\}}^{-\frac{1}{2}}=\sqrt{\sigma^{2}\,/\,I}$$

**FIGURE 3:**
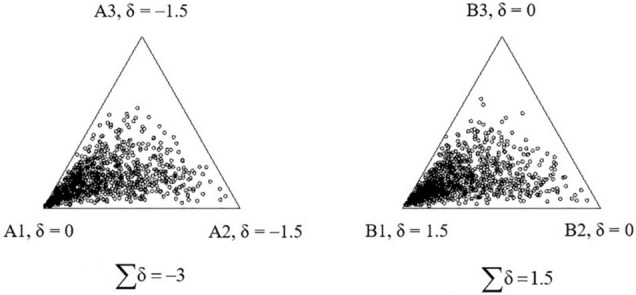
The 1,000 replications of a single person’s responses to the two compositional items, δ_*A*_ = [0, −1.5,−1.5] and δ_*B*_ = [1.5, 0, 0], under the lognormal framework.

The standard error is constant regardless the values of θ and δ. To illustrate the standard error in the compositional responses, Figure 3 shows 1,000 replicated responses of a single person with θ = [0, 0, 0] answering item A (left) with **δ** = [0, −1.5, −1.5] and item B (right) with **δ** = [1.5, 0, 0]. For both items, the parameter of the first statement is 1.5 larger than the parameters of the second and third statements. The closer the dot is to the statement (e.g., statement A1), the higher the score given by the person to the statement. For both items, the first statement (A1 or B1) has higher utility than the other two statements. Therefore, the first statement is generally more attractive and is endorsed more often than the other two statements, and the person tends to give a higher expected score to the first statement than to the second or third statement. As expected, the numerous replicated responses are close to the first statement (Figure 3). In addition, the standard error is constant over items in the LIM, so that the distribution of the replicated responses is similar for items A and B.

### Explanation of Point Estimates

The proposed model retains the nature of ipsative scores, that is, the sum of scores within persons is constant. The comparison between persons can be made in terms of aspects of the profile or of differentiation, and not of individual traits. The profile aspect means that the explanation is in terms of the pattern of latent traits for each person. The differentiation aspect means that the explanation is made in terms of the range of latent traits for each person. For example, one person’s personality scores may have a range of 3.0, which is larger than a range of 0.5 in another person’s personality scores.

As the explanation of compositional data is based on a person’s profile, that is, how the dimensions for the person are differentiated, the scores are measured at a ratio level. The *zero point* of the ratio scale in the compositional data should represent the non-differentiation across dimensions, and in this case, the scores for all dimensions are identical. For example, in the four-dimensional tests, a person has four equal raw scores, such as [0.25, 0.25, 0.25, 0.25]. This means that the differentiation between the four scores for this person is zero. In the LIM, the non-differentiation gives the latent traits of [0, 0, 0, 0] because the within-person mean is constrained to zero. A positive value indicates that the latent trait is higher than the within-person mean, and a negative value indicates that the latent trait is lower than the within-person mean.

### Imputation for Zero Response

Note that a zero response causes the convergence problem in parameter estimations. In the LIM, the log ratio *Y*_*kD*_, including zeros, yields either negative infinity (when *X*_*k*_ = 0, log(*X*_*k*_/*X*_*D*_) = −∞) or positive infinity (when *X*_*D*_ = 0, log(*X*_*k*_/*X*_*D*_) = ∞). One effective solution to the problem of zero responses is to use the imputation method (Martín-Fernández et al., 2003), through which any zero can be replaced with a fixed imputed value κ, which is a pre-specified value smaller than the possible smallest response value. For example, when *C* = 100 and the smallest point that can be responded to any one statement is 1.0, the imputation method can be used to replace all of the zero responses with a constant value of κ and 0 < κ < 1.0 (e.g., κ can be 0.5). Note that the replacement of zero distorts the fixed sum of *C*. In an item, a replacement of zero with κ changes *C* into *C*+κ. Therefore, nonzero responses must be adjusted to preserve the fixed sum of *C*. The imputation method with adjustment is as follows:

(16) $$X_{k}=\left\{ \begin{array}{ccc} \kappa \, & ,\text{if} & X_{k}=0\\ X_{k}\,\left(1-\frac{1}{\text{C}}\,\sum_{X_{k^{\prime }}=0}\kappa \right) & ,\text{if} & X_{k}\neq 0 \end{array}\right.$$

Martín-Fernández et al. (2003) suggested that the imputed value *κ* = 0.65 of the smallest possible response performs best when the proportion of zero in the data is below 10% of the total number of elements in the data set (Martín-Fernández et al., 2003). Brown (2016b) recommended using *κ* = 0.5 to achieve the minimum mean square Aitchison distance for psychological and educational data. The current study adopted the suggestion of *κ* = 0.5.

### Model Comparison: Lognormal Ipsative Model Versus Thurstonian Model for Compositional Items

Both the TMC (Brown, 2016b) and the LIM employ the additive log ratio transformation (Aitchison, 1982), in which *D* raw compositional data are transferred to *D*−1 log ratio data (i.e., *D*−1 components divided by the reference component and logarithms taken). Comparing Eqs. 3 and 5, the TMC is a two-parameter model with a slope parameter for each statement, whereas the LIM is a one-parameter model that only considers the statement utility (location parameter). Mathematically, LIM is a special case of the TMC. Nevertheless, this section presents the fundamental differences between them.

The LIM and the TMC serve different measurement purposes. The TMC aims to recover person parameters that represent normative latent traits that can be compared (i.e., a single trait by a single trait) between persons. By contrast, the LIM aims to obtain the measures with the property of specific objectivity, to recover the person parameters always when all statements using positive keys, and to converge all the time. A model with the good parameter-recovery in equally keyed statement design and achieves a perfect convergency rate would be highly desired characteristics for practitioners who may face the convergence problem in the application of TMC.

The TMC yields utilities (location parameters) for pairs of statements, whereas the LIM yields the unique utility for each individual statement. As can be seen in Eq. 3 for TMC, only δ*_*k_D*_* is obtained as the utility for the pair of statements *k* and *D*. The LIM has the unique utility δ*_*k*_* and δ*_*D*_* (Eq. 5) regardless of the use of compositional items.

The drawback of using the LIM is that it maintains the ipsative nature of the constant sum of latent traits such that the test users must explain the test scores using the ipsative way (see the section of “Explanation of Point Estimates”). The between-person comparison of scores on a single dimension could not be made. With the TMC, although test user can explain the scores in the normative way, there is always the risk of bias in the latent trait estimates in the condition of equally keyed statement design and the difficulty of the convergence problem, even when the response data perfectly follows the TMC (Bürkner et al., 2019).

### Evaluation of Model Fit

This section introduces the model fit diagnostic methods used to examine the data fit for the LIM. The PPMC method for compositional data was adopted in the data analysis. PPMC works under the Bayesian theorem. Let π denote the parameters in the model, that is, either person ability or statement utility. *P*(π) is the prior distribution for parameter π. The posterior distribution combines the information of the *P*(π) and the information in the observed data **y**_*obs*_, which can be obtained using Bayesian probability:

(17) $$P\,\left(\pi |{\text{y}}_{\text{obs}}\right)=P\,\left({\text{y}}_{\text{obs}}|\pi \right)\times P\,\left(\pi \right)\,/\,P\,\left({\text{y}}_{\text{obs}}\right)\propto P\,\left({\text{y}}_{\text{obs}}|\pi \right)\times P\,\left(\pi \right)$$

where *P*(π | **y**_*obs*_) is the posterior distribution of the parameter π given the observed data **y**_*obs*_, and *P*(**y**_*obs*_ | π) is the likelihood function of the fitted model. In PPMC, the posterior can be used to draw the replicated data, **y**_*rep*_. As **y**_*rep*_ is generated based on the parameter π of the fitted model, it implies a prediction of the response data if the model is true. To assess the model fit, the discrepancy statistic ξ, which is a function of the data set **y**, is chosen. ξ(**y**_*obs*_) is the discrepancy statistic of the observed data, and ξ(**y**_*rep*_) is that of the replicated data. PPMC demonstrates a poor model–data fit when the value of ξ(**y**_*obs*_) is out of the credible interval of ξ(**y**_*rep*_) distribution, whereas it shows a good model–data fit when the value of ξ(**y**_*obs*_) is within the credible interval of ξ(**y**_*rep*_) distribution (Meng, 1994). Therefore, to measure the model fit, Prob[*ξ* (*y*_rep_) > *ξ* (*y*_obs_)]is calculated, denoted by *pr*. The estimate *pr* can be obtained as follows:

(18) $$pr=\frac{1}{T}\sum_{t=1}^{T}\text{I}\left(\text{ξ}\left({\text{y}}_{\text{rep}}^{\left(t\right)}\right)\geq \text{ξ}\left({\text{y}}_{\text{obs}}\right)\right)$$

where *T* is the number of replications, $\text{ξ}\,\left({\text{y}}_{\text{rep}}^{\left(t\right)}\right)$ is the tested statistic computed from the *t*th replication of **y**_*rep*_; and *I*(.) is an indicator function, which is equal to 1 when ξ(**y**_*rep*_) ≥ ξ(**y**_*obs*_) is true, and 0 otherwise. Generally, a *pr* smaller than 0.025 or larger than 0.975 suggests a model misfit (i.e., nominal type I error rate equal to 0.05; Meng, 1994).

The sum of the profile differentiation can be chosen as the discrepancy statistics in this study because the purpose of compositional items is to measure the profile distribution of the traits within persons. A person *n*’s profile differentiation is defined as the absolute difference between his/her highest and lowest raw scores (O’Neil, 1977) and is expressed as follows:

(19) Differentiation=max⁡(1⁢Y)-min⁡(1⁢Y)

where **Y** is a *I* × *D* matrix indicating person *n*’s *D*-dimensional observed scores to *I* items, and **1** is a 1 × *I* row-vector with all elements equal to one. The observed profile differentiation across persons located within the 95% credible interval of the replicated profile differentiation based on the posterior distribution represents an acceptable model–data fit.

## Empirical Study Using an Online Value Test in a Compositional Format

To illustrate the application and implication of LIM, we created an online test using compositional items to collect real responses and analyzed the collected compositional data using both the LIM and the TMC. This empirical study included the development of an online value test using compositional items, real data collection, and data analysis.

### Materials

An online value test based on Schwartz’s values theory with compositional items was developed. A total of 32 statements were modified from the World Values Survey Online (Inglehart et al., 1998), the contents of which I rewrote to suit the compositional format. According to Schwartz’s value theory (Schwartz, 1994; Schwartz and Boehnke, 2004), these statements measure four different dimensions of values: Self-transcendence, Conservation, Self-enhancement, and Openness to Change. According to Schwartz’s (1994) framework, Self-transcendence emphasized the acceptance of others as equal and concern for their welfare; Conservation indicates the extent of a person’s stance toward classical liberalism; Self-enhancement is related to persons who emphasize the pursuit of their own relative success and dominance over others; Openness to Change is related to people who emphasize independent thought and action and favor change.

Each of the four dimensions was measured by 8 of the 32 statements. Statement numbers 1–8 measure Self-Transcendence, 9–16 measure Conservation, 17–24 measure Self-Enhancement, and 25–32 measure Openness to Change. Based on the partial linkage design, we developed 40 compositional items. The linkage design (assignment of statements to items) of this survey is presented in Table 1. The instructions in the items stated the following: “Please allocate 100 points to indicate how important the descriptions of these values are in your life. Give a higher number of points to the statement that is more important to you.” Besides the values measured in the online value test, demographic variables were collected in the survey, including gender, age, education level, and religion. The contents of the test are given in the Appendix B. The items were uploaded to the online survey service QuestionPro^1^.

**TABLE 1:** Statement numbers in the online value test with compositional items.

Item number	ST	CS	SE	OC	Item number	ST	CS	SE	OC
1	1	9	17	25	21	2	9	24	31
2	2	10	18	26	22	7	10	17	32
3	3	11	19	27	23	8	15	18	25
4	4	12	20	28	24	1	16	23	26
5	1	10	19	28	25	1	11	21	31
6	2	11	20	25	26	3	13	23	25
7	3	12	17	26	27	5	15	17	27
8	4	9	18	27	28	7	9	19	29
9	5	13	21	29	29	2	12	22	32
10	6	14	22	30	30	4	14	24	26
11	7	15	23	31	31	6	16	18	28
12	8	16	24	32	32	8	10	20	30
13	5	14	23	32	33	1	12	23	26
14	6	15	24	29	34	2	13	24	27
15	7	16	21	30	35	3	14	17	28
16	8	13	22	31	36	4	15	18	29
17	6	13	20	27	37	5	16	19	30
18	3	14	21	28	38	6	9	20	31
19	4	11	22	29	39	7	10	21	32
20	5	12	19	30	40	8	11	22	25

*ST, Self-Transcendence; CS, Conservation; SE, Self-Enhancement; and OC, Openness to change.*

### Participants and Sampling

Convenience sampling was used to administer the surveys. We requested student helpers to complete the test and distribute the survey website link to their peers. Seven student helpers who were enrolled in an undergraduate degree program at the Education University of Hong Kong at the time of the study were hired. Each helper was requested to distribute the survey link to at least 60 friends. The total number of participants was 577 persons aged 12–52 years. The sample comprised 190 males and 387 females.

### Data Analysis

In the data preprocessing, zero responses were assigned a constant value of 0.65 in accordance with Martín-Fernández et al.’s (2003) recommendation. We ensured that all the responses were reasonable by screening and reflecting upon each returned survey. For example, nonsense responses, such as allocating 100 points to the first statement in all items, were eliminated from the data set (65 cases). Further, we found that several respondents had completed the survey very quickly, which might indicate a low motivation to complete the survey. Randomly responding to items without paying attention to the item contents translated to a survey completion time of about 250 s. By removing from the sample those that had response times below 300 s (too fast) or the aforementioned nonsense responses, the remaining data set for further analysis comprised a sample of 512 respondents. The distributions of the respondents’ demographic variables are presented in Table 2.

**TABLE 2:** Demographic variables of the respondents in the analyzed samples.

Gender		Religion	
Male	167	Catholic	98
Female	345	Islam	5
		Hinduism	7
		Chinese tradition	24
*Age*		Buddhism	15
11∼15	4	No Religion	361
16∼20	232	Other	2
21∼25	255		
26∼30	13	*Education Level*	
31∼35	3	Elementary	3
36∼40	1	High School	24
41∼45	1	Undergraduate	462
46∼50	2	Postgraduate	15
51∼	1	Other	8

After data cleaning, the real data collected from the survey were fitted to the LIM and the TMC. To apply the TMC approach and replicate Brown’s (2016b) work, we fitted the TMC using maximum likelihood estimation in the software package M*plus* (Muthén and Muthén, 1998–2012). Unfortunately, M*plus* reported the massage of “The standard errors of the model parameter estimates could not be computed. The model may not be identified.” Apparently, the TMC was not identified when the matrix of factor loadings was not fully ranked. This observation is consistent with the notes by Brown (2016a). We then fitted the LIM and TMC by using MCMC algorithm that proposed in this study.

The MCMC algorithm was used for parameter estimation and implemented using the JAGS software (Plummer, 2017). The JAGS syntax for LIM can be found in Appendix C. The prior distribution of the statement utilities was set to *N*(0, 1). The prior for the latent traits was specified to follow the multivariate normal distribution with a mean of zero and a variance of one. The prior for the covariance between latent traits was set as an inverse Wishart distribution [**R**, *K*] with **R** = ***I*** and hyperparameter *K* = 3. All prior distributions were set the same with the descriptions in the section of parameter estimation. Two chains of the MCMC were generated. The mean of the samples from the posterior distribution in the 10,000 iterations after the 10,000 burn-in iterations in the MCMC was defined as the point estimate. The estimation of the MCMC facilitated the analysis of the PPMC, as the samples in the MCMC could be directly used as the replicated data **y**_*rep*_.

The convergence of the MCMC estimation was examined by tracing the posterior sampling of the parameters. We calculated the potential scale reduction factor (PSR; Gelman and Rubin, 1992), which is the typical index used to assess the convergence of the MCMC. This index is the ratio of the credible interval between the total sequence and the mean of the within-sequence in the MCMC sampling. A PSR value that is closer to one means that the model estimation converges well (Gelman and Rubin, 1992). We expected that the TMC couldn’t obtain the converged result because the non-full rank of factor loading problem should not be resolved by changing the estimator from the maximum likelihood method to the MCMC algorithm.

One purpose of the empirical study is to present an example of the practical interpretations of the results under the proposed model. To achieve this, the descriptive analysis of the estimates of a person’s latent traits and statement utilities for the proposed model are presented first, and then the correlations between the raw scores and the latent trait estimates in the LIM are shown to give an idea of the latent traits from the proposed model. We selected two persons to illustrate the values of the latent traits and their meanings in practice.

The reliability statistics were calculated. The variance of the latent trait estimates and their standard errors were used to calculate reliability. The population error variance of each dimension was obtained by the squared standard error. In accordance with the classic definition that the proportions of variance in the intended traits are accounted for by the true score, reliability was calculated as follows:

(20) $$\rho =v\,a\,r\,\left(\theta \right)/v\,a\,r\,\left(\hat{\theta }\right)=\left[v\,a\,r\,\left(\hat{\theta }\right)-S\,E^{2}\,\left(\hat{\theta }\right)\right]\,/\,v\,a\,r\,\left(\hat{\theta }\right)$$

The LIM was expected to have reliabilities above the acceptable level of 0.7.

### Results of the Empirical Study

**FIGURE 4:**
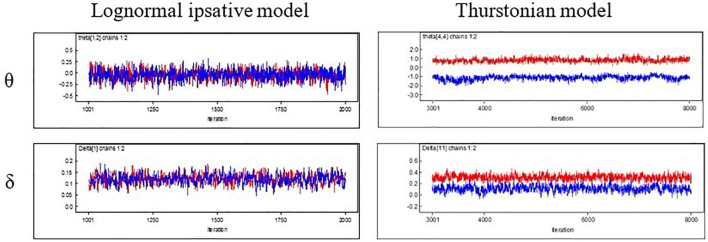
Trace of posterior sampling for the two models in MCMC.

Four aspects of the results are presented and discussed in this section: (1) convergence of the MCMC, (2) estimates of the statement utilities, (3) correlation between the latent trait estimates and the raw scores, and (4) model–data fit statistics of the new model. The convergence of the MCMC was evaluated first. Figure 4 presents the trace of the posterior sampling for person estimates (upper) and utility estimates (lower) in both the LIM (left) and the TMC (right). To the LIM, the values of each estimate for the sampling sequences stabilized at a small range. This result indicates that the MCMC estimation converges well. Similar results were observed for the PSR index, with values of around 1. The PSR result for each parameter was within the range of 0.998–1.002 in the LIM. The TMC, unfortunately, failed to converge when fitting to this dataset (see the trace of posterior sampling in Figure 4). Consequently, the results below are only for the LIM.

**FIGURE 5:**
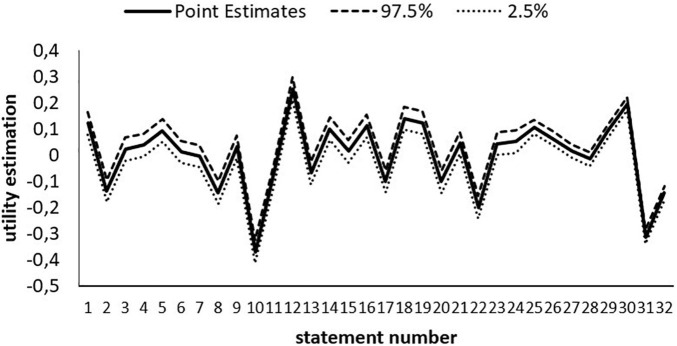
Utility estimates of the statements at the 97.5 and 2.5% quantiles.

The results of the statement utility estimates are reported. Figure 5 shows the 32 statement utility estimates. Statement numbers 12 (“To be polite to other people all the time. Never disturb or irritate others”) and 30 (“I really want to enjoy life. Having a good time is very important to me”) have the highest utilities, whereas statement numbers 10 (“It is best to do things in traditional ways. To follow the customs or religions that we have”) and 31 (“I like to take risks. I am always looking for adventure”) have the lowest utilities.

**FIGURE 6:**
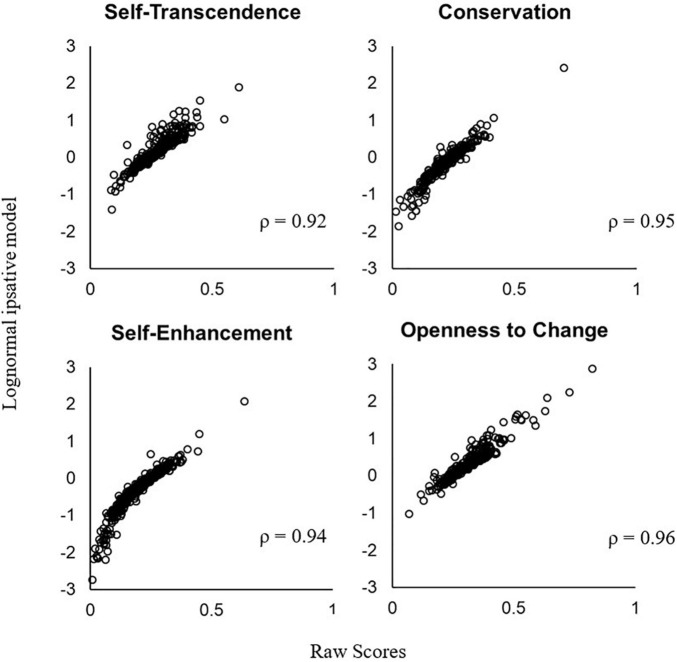
Relationship between a person’s raw score and trait estimate in the lognormal ipsative model.

The correlations between the latent trait estimates and the raw scores are 0.92 for Self-Transcendence, 0.95 for Conservation, 0.94 for Self-Enhancement, and 0.96 for Openness to Change (Figure 6). A person with higher raw scores also generally has higher latent traits in the LIM. However, the raw scores and the latent traits in the LIM obviously lie on different scales (a non-linear relationship). The LIM uses the log ratio transformation and makes a person’s θs sufficient for specific objectivity, which the raw scores do not satisfy.

Table 3 shows the means, standard deviations, and correlations between the four measures in the raw score and in the LIM. The raw scores range from 0 to 1 by its definition. The four measures have the means of [0.27, 0.22, 0.21, 0.30] and the standard deviations of [0.06, 0.06, 0.07, 0.07]. The inter-trait correlation ranges from −0.60 to −0.01. In the LIM, the means of the four latent traits are [0.13, −0.13, −0.26, 0.26], and the standard deviations are [0.34, 0.36, 0.51, 0.37]. The correlation between the four traits ranges from −0.74 to −0.05 in the LIM. As expected, the compositional data yield negative correlations on the person traits because of the ipsative constraint (i.e., the sum of the responses within an item is set to equal a constant). The inter-trait correlation in the lognormal ipsative model is similar to that in the raw score, implying that the factor structure does not change when using the LIM. One controversy is that practitioners can even use raw scores for this purpose without the need of fitting a complex IRT model in the presence of similar simple structure between traits. However, raw scores do not satisfy the specific objectivity and do not reference the item parameters. The LIM established the scores on the scales, regardless of the change of statements in the test, as a measurement property in Rasch model.

**TABLE 3:** Summary of the means, standard deviations, and correlations of the four measures in the raw score and in the lognormal ipsative model.

			Correlations		
Measure	Mean	SD	1	2	3
*Raw Score*					
1. Self-Transcendence	0.27	0.06	−		
2. Conservation	0.22	0.06	−0.01	−	
3. Self-Enhancement	0.21	0.07	−0.60	−0.20	−
4. Openness to change	0.30	0.07	−0.21	−0.59	−0.35
*Lognormal ipsative model*					
1. Self-Transcendence	0.13	0.34	−		
2. Conservation	−0.13	0.36	−0.05	−	
3. Self-Enhancement	−0.26	0.51	−0.74	−0.27	−
4. Openness to Change	0.26	0.37	−0.16	−0.56	−0.44

To illustrate the meaning of the latent traits, we took person no. 003 as an example. Using the estimates in the LIM, person no. 003 has the highest estimated value (1.22) for Self-Transcendence among the four traits. This means that he/she prefers to help others, loves nature, and believes in protecting the weakest members of society. However, he/she has the lowest estimated value (−1.52) for Self-Enhancement among the four traits. This finding indicates that he/she places less importance on emphasizing his/her success and dominance over others.

By contrast, person no. 410 is an example of low differentiation. His/her four trait values are close to each other. In the LIM, person no. 410 has the vector of latent traits **θ** = [−0.08, 0.16, −0.21, 0.13]. Although he/she values Conservation the highest (0.16) and Self-Enhancement the lowest (−0.21), both latent traits are close to zero (the range between the two values is 0.37). This result indicates that all four values are equally important to him/her.

To assess the model–data fit, the PPMC method was employed. The results show that the model provides a good model–data fit. The observed sum of the profile differentiation across persons located at the 30.7th percentile of the replicated sum of the profile differentiation. It is located within the 95% credible interval; therefore, the proposed model has a good model–data fit according to the criterion established in the methodology section. The error variance estimates is 0.087. The reliabilities of the four dimensions in the LIM are 0.85, 0.86, 0.93, and 0.96. All reliabilities are higher than 0.85.

## Simulation Studies

The simulation study aims to investigate the precision of the parameter estimation for both the LIM and the TMC when the response data followed the LIM, which is a special case of the TMC with all factor loadings fixed to one and ipsative constraints. It means the TMC definitely met the non-full rank of factor loading problem in our simulation. Since the TMC often fails to converge in this special case, the comparison of convergency rate between the two models is also presented in the simulation results.

### Parameter Generation

A four-dimensional test using compositional items with four statements in different dimensions was conducted. The statement utility parameters were generated from −1.2 to +1.2 following a uniform distribution. The distribution of statement utilities corresponded to the result of empirical data analysis in this study. For identification, the sum of the utilities within the items was set to zero. The test length was manipulated in three different conditions, 10 items, 20 items, and 40 items.

To evaluate the effect of the sample size, we manipulated the sample sizes of 250, 500, and 1,000 persons. The persons were generated with four normative latent traits [θ_1_, θ_2_, θ_3_, θ_4_] following a multivariate normal distribution with means of [0, 0, 0, 0], and all standard deviations set to equal one. The correlation between latent traits was manipulated into six conditions: all correlations set to (1) 0.8, (2) 0.5, (3) 0.2, (4) 0, (5)–0.2, and (6) a real-world correlation matrix (Bürkner et al., 2019). The real-world correlation matrix is [1, −0.33, −0.43, −0.37; −0.33, 1, −0.30, 0.32; −0.43, 0.30, 1, 0.27; −0.37, 0.32, 0.27, 1]. The conditions of correlations to 0.8, 0.5, and 0.2 represents the strong, mediate, weak positive intercorrelations. The two conditions of correlations to 0 and −0.2 represent no intercorrelation and negative intercorrelation, respectively. To create the ipsative scores from the normative scores (i.e., constraint of θ_1_ + θ_2_ + θ_3_ + θ_4_ = 0 for each person), we subtracted the within-person mean of the latent traits from the person’s generated latent traits as the true person parameters.

The response data sets were generated by the LIM and replicated 100 times using the R2jags package (Su, 2015) in R software. The TMC and LIM were used to fit to the corresponding data sets. An MCMC estimator the same to the empirical study was used as the estimation method in the simulation study.

To evaluate the recovery of item and person parameters, bias and the root mean square error (RMSE) of the estimates were employed and computed as follows:

(21) $$\text{Bias}=\sum_{\text{t}=1}^{T}\left({\hat{\pi }}_{\text{t}}-\pi_{\text{t}}\right)\,/\,T,\text{and}\,\text{RMSE}=\sqrt{\sum_{\text{t}=1}^{T}{\left({\hat{\pi }}_{\text{t}}-\pi_{\text{t}}\right)}^{2}\,/\,T}$$

where *T* is the number of replications (*T* = 100), $\hat{\pi }$ is the estimates of the parameters, and π is the true value of the parameters. All estimates of the statement and person parameters (δ and θ) could have bias and RMSE across replications. The estimation of correlation between latent traits $\sigma_{\theta_{d}\,\theta_{d^{\prime }}}$ which represents the structural framework between the measures was evaluated by the relative bias $\sum_{t=1}^{T}\left({\hat{\pi }}_{\text{t}}-\pi_{\text{t}}\right)\,/\,\left(T\times \left|\pi_{\text{t}}\right|\right)$ and relative absolute bias $\sum_{t=1}^{T}\left|\left({\hat{\pi }}_{\text{t}}-\pi_{\text{t}}\right)\,/\,\left(T\times \pi_{\text{t}}\right)\right|$.

In summary, this simulation experiment had 3 × 3 × 6 = 54 unique conditions across three test length conditions (i.e., 10, 20, and 40 items), four sample-size conditions (i.e., 250, 500, and 1,000 persons), and six intercorrelation conditions (i.e., 8, 0.5, 0.2, 0, −0.2, and a real-world correlation matrix). The bias and RMSE in Eq. 21 were averaged within the conditions, so that the means of the bias and RMSE were compared between conditions.

### Results of Simulation Study

**FIGURE 7:**
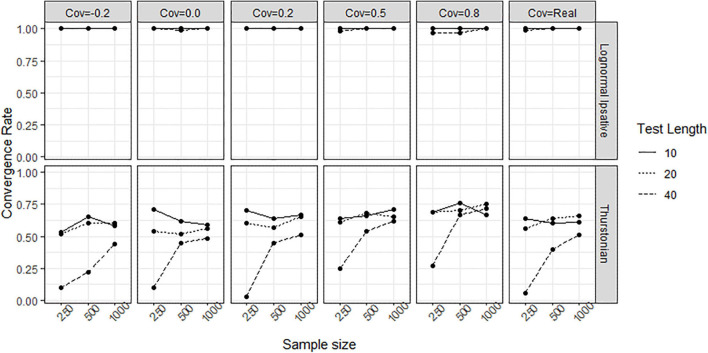
Convergence rate obtained by fitting the lognormal ipsative model and the Thurstonian model to simulated data. The types of lines indicate the test lengths. Cov = the covariance between latent traits. Cov = Real indicates the variance-covariance matrix followed the empirical result in literature.

In simulation study, tests with different test lengths and sample sizes, and the covariances between latent traits following different scenario were manipulated to form different conditions. Figure 7 shows the convergence rate of the LIM (upper plots) and the TMC (lower plots) in the different conditions. During the 100 replications, the LIM converged well across conditions with convergence rates of greater than 97%, whereas the TMC failed to converge especially under the condition of 40 items (long test length) and 250 persons (low sample size). The higher covariance matrix between traits increased the convergence rate for the TMC.

**FIGURE 8:**
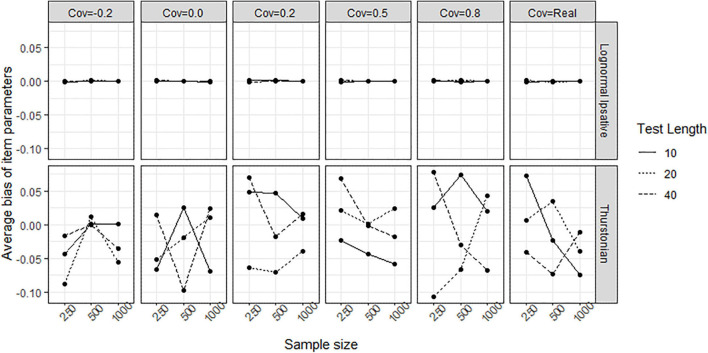
Average bias of item parameters obtained by fitting the LIM and the Thurstonian model to simulated data. The types of lines indicate the test lengths. Cov = the covariance between latent trait. Cov = Real indicates the variance-covariance matrix followed the empirical result in literature.

**FIGURE 9:**
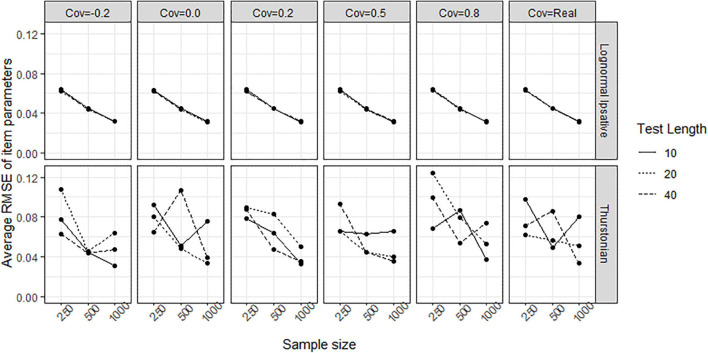
Average RMSE of item parameters obtained by fitting the lognormal ipsative model and the Thurstonian model to simulated data. RMSE is root mean square errors. The types of lines indicate the test lengths. Cov = the covariance between latent trait. Cov = Real indicates the variance-covariance matrix followed the empirical result in literature.

Figure 8 shows the average biases of item parameter estimation averaged over all items in all conditions for both models. The LIM (upper plots in Figure 8) had bias close to zero in all conditions, whereas the TMC (lower plot in Figure 8) had serious biases in all conditions. The extent of the bias seems not to be related to test length, sample size, and covariance between latent traits. Figure 9 shows the average RMSEs of item parameters estimation in the different conditions for both models. In the LIM (upper plots in Figure 9), the larger the sample size the lower the RMSE obtained. The different test lengths and the covariances between latent traits did not change the RMSE of item estimation. The TMC (lower plots in Figure 9) always had the a higher RMSE than the LIM across all the conditions. Increasing sample size leads generally to a decline in the RMSE. The change of RMSEs seems not related to the conditions of test lengths and covariances between traits.

**FIGURE 10:**
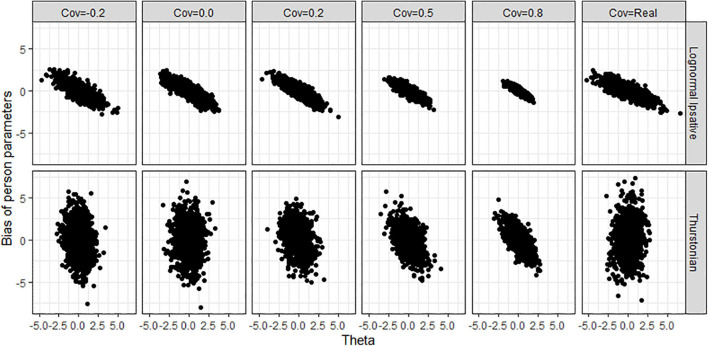
Bias of person parameters obtained by fitting the lognormal ipsative model and the Thurstonian model to simulated data. Cov = the covariance between latent trait. Cov = Real indicates the variance-covariance matrix followed the empirical result in literature.

**FIGURE 11:**
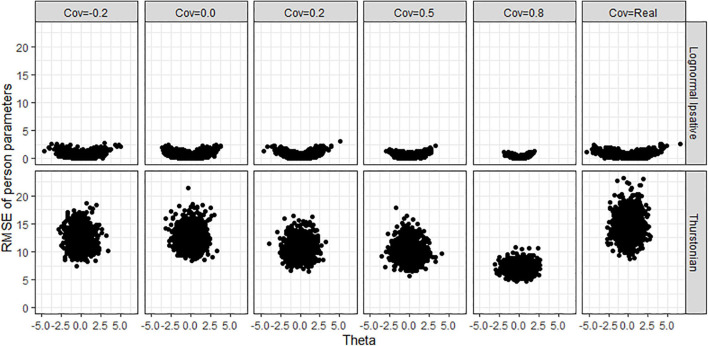
RMSE of person parameters obtained by fitting the lognormal ipsative model and the Thurstonian model to simulated data. RMSE is root mean square errors. Cov = the covariance between latent trait. Cov = Real indicates the variance-covariance matrix followed the empirical result in literature.

Figure 10 shows the biases of latent traits estimation by the trait levels (*x*-axis) in various covariance matrices for both models when the condition is 40 items and 1,000 persons. The different test lengths and sample size did not change the shape of the scatter plots so for the reasons of simplicity, we do not show plots for them. LIM (upper plots in Figure 10) had negative biases in high trait-level persons and positive biases in low trait-level persons. This shrinkage of person estimation probably resulted from the prior distribution in Bayesian estimation. The TMC (lower plots in Figure 10) had a large range of bias across trait-levels. Figure 11 shows the RMSEs of latent traits estimation by trait-levels for both models. The TMC (lower plots in Figure 11) produced huge RMSEs indicating that the standard error of the person estimation was extremely large, especially when the variance-covariance matrix followed the real matrix. On the contrary, the LIM (upper plots in Figure 11) produced reasonable RMSEs.

**FIGURE 12:**
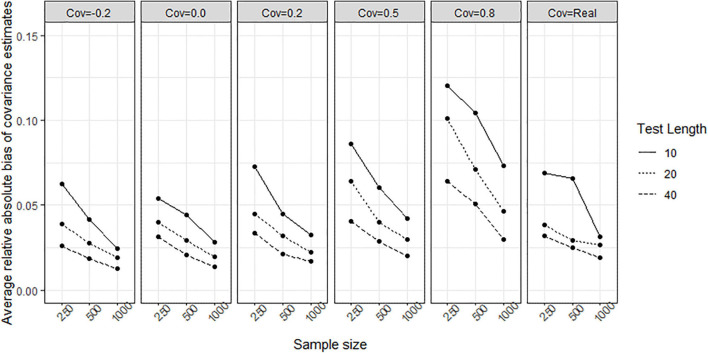
Average relative absolute bias of variance-covariance estimation obtained by fitting the lognormal ipsative model to simulated data. Cov = the covariance between latent trait. Cov = Real indicates the variance-covariance matrix followed the empirical result in literature.

To evaluate whether the structure among traits change when fitting the models, the relative biases and relative absolute biases of variance-covariance estimation for both models was observed. For the TMC, the relative bias ranged from −505.901 to 434.817, and the relative absolute bias ranged from 9.431 to 525.972. This implies that (1) the TMC converges to extremely wrong values and (2) TMC fails to recover the structure between traits. For the LIM, the relative bias ranged from −0.013 to 0.008 across all conditions. The relative absolute bias decreased when sample size increased, test length increased, and covariance was close to zero (see Figure 12). All relative absolute bias across conditions were lower than 0.12. The structure among traits in the LIM were recovered.

## Discussion and Conclusion

In this study, we developed a new model called the LIM for analyzing compositional items, overcoming the limitations of TMC (Brown, 2016b), which is the only model having been used in IRT to examine compositional items, but even TMC has not considered the model’s violation of specific objectivity. Most importantly, parameter estimates in the TMC are strongly biased in the condition of all equally keyed statements. In that case, this model suffers from convergence problems because of the failure of the model identification. The current study addresses this problem directly by developing a new IRT model that satisfies the conditions of specific objectivity, unbiased estimation in test design with equally keyed statements, and a convergence rate close to 100% for the analysis of compositional items. The simulation studies showed that the parameters in the proposed model could recover well the values in the simulated data using MCMC estimation.

This research made use of an online value survey comprising 40 compositional items that were developed according to Schwartz’s value theory to ascertain the applicability of the LIM, newly developed for the analysis of compositional data in empirical settings. The response data set used a sample size of 512 individuals, whose responses were analyzed by the proposed model since the TMC failed to be converged. The examination of the model–data fit through the PPMC method showed that the LIM had an acceptable model–data fit. The reliabilities were greater than 0.85 in the model.

When response data generated from LIM in which all items have equal keyed statements, the TMC had worse convergence rate than the LIM, especially in the condition of small sample size (250 persons) and short test length (10 items). The item parameters and person parameters were biased estimated in TMC. The LIM had the convergence rate close to 100% across conditions of different test lengths, sample sizes, and covariance between traits. It implies that using TMC in an all-equal-keys situation takes the risk of non-converged result in the model estimation which has been concluded by Bürkner et al. (2019). The LIM successes to overcome this problem.

The precision of item parameter estimation increases as the sample size increases. The precision of person parameter estimation increases as the test length increases. The precision of covariance between traits rises when the test length and sample size increase. Those findings corroborate the previous results in IRT modeling for ipsative data (Brown and Maydeu-Olivares, 2011; Wang et al., 2017).

The high precision of parameter estimates obtained in the simulation study has demonstrated that the proposed model allows the practitioners to develop a compositional test with an equally keyed statement design which cannot be allowed in using TMC because of the biased estimation and convergency problem. Using tests containing equally keyed statements will help avoid many of the problems encountered when using negatively keyed statements. The first problem is the dimensionality problem, in which the two oppositely keyed statements (positively and negatively) imply different underlining factors (Dueber et al., 2018). The second issue concerns how the negatively keyed statement undermines the forced-choice items’ advantage in the resisting the problems of *faking good responses* and *the social desirability effect* (Bürkner et al., 2019). Furthermore, using the negatively keyed statement might increase the cognitive load placed on the test taker. It implies that the test result might rely on the test taker’s working memory capability and limit the potential construct validity of the test. Therefore, the LIM enables test developers to avoid the use of negatively keyed statements and circumvents the associated problems.

Compared with the existing TMC by which the test takers can obtain their scores on a normative scale, the drawback of the new model is that it yields scores in an ipsative way, where the sum of scores across traits within persons is constrained to zero. Practitioners would prefer not to obtain test scores that only represent the differentiation or the relative locations between traits (i.e., explanation of ipsative scores) when they need to rank test takers by their scores for individual traits (i.e., explanation of normative scores). The between-person comparison in using ipsative scores, if required by practitioners, should be based on the person’s differentiation rather than scores on a single trait. In other words, in using the LIM, ranking test takers is only allowed in terms of the differentiation among traits. In choosing between the TMC and the LIM for analyzing compositional item response data there is a trade-off between avoiding the problems associated with the TMC, vis-à-vis convergence and estimation biases (in equal keys design tests), and the challenge associated with explaining ipsative scores in the LIM. At least, when the TMC has failed to converge in fitting a data, the LIM provides a solution for practitioners.

The LIM sacrificed the chance of generating the normative scores as the TMC did, but had specific objectivity. Of course, specific objectivity is not the “Holy Grail” of scale properties and, in fact, is inappropriate for the measurement purpose of Thurstonian IRT models when creating the normative scores for ipsative tests with forced-choice items. Specific objectivity would not be a big issue in using the TMC for compositional response data. This study does not reject using TMC – instead, it provided an alternative of the measurement model for compositional items other than the TMC as an option for practitioners.

In summary, key advantages of the LIM are the feature of specific objectivity and the possibilities to overcome the convergence problem in modeling compositional data. Nevertheless, several limitations of the LIM should be noted here: First, LIM does not allow a unidimensional structure. This limitation matches Brown’s conclusion that when the number of the dimension is one (*D* = 1) and all factor loadings are constant, the latent variable is not informative. Second, the LIM did not generate the normative scores that TMC did. Practitioners might wish to obtain the normative scores from the ipsative data structure – however, the LIM did not generate these scores.

Moreover, our study has some limitations: First, no psychological theory has yet been proposed that supports the necessity for using compositional items in tests. The issue of whether a forced-choice format can avoid the effect of social desirability has been explored and reported in the literature (Meade, 2004). Nevertheless, ranking items and the pairwise comparison of items are easier to apply in practice than compositional items because responding to categorical options for these items is easy in paper-and-pencil tests. Even with the rapid development of computerized tests, a continuous response format is expected to gain more popularity in the future, as there is a lack of theoretical reasons to use compositional items in psychological tests. Second, the data sample that I analyzed might not be representative of humans in general. My convenience sample was drawn only from people in Hong Kong who were friends with undergraduate students enrolled at The Education University of Hong Kong. The relatively restricted sample may limit the generalizability of the results of this study.

As a recommendation for future studies, the application of multilevel models is useful in educational research. The multilevel model takes into account the nested data structure in the modeling process. For example, the method of the Program for International Student Assessment is to sample the first the schools and then the students nested within the schools. Skrondal and Rabe-Hesketh (2003) proposed a framework for multilevel modeling of ranking data engaging the covariates at the different levels. Future research can explore the application of the LIM to the multilevel structure of tests involving compositional data.

The proposed LIM is a dominance model. As of today, probabilistic models for unfolded ipsative and continuous data have not yet been reported in the IRT literature. Unfolded response means that the respondents are expected to have higher scores for statements in which their latent trait values are closer to the utility. To model the continuous ipsative response data in the future, we suggest that, given the LIM, the ideal point concept for the probability of the response to statement *k* in a *D*-dimensional compositional item can be written as

(22) $$Y_{k\,\_\,D}={\left(\theta_{D}-\delta_{D}\right)}^{2}-{\left(\theta_{k}-\delta_{k}\right)}^{2}+\varepsilon_{k\,\_\,D}$$

where *Y*_*k_D*_ is the log ratio of *X*_*k*_ to *X*_*D*_ (the response to statement *k* and the response to statement *D*); θ*_*k*_* and θ*_*D*_* are the person’s latent traits on dimensions *k* and *D*, respectively; δ*_*k*_* and δ*_*D*_* are the utilities of statements *k* and *D*, respectively; and ε*_*kD*_* is the error term following the normal distribution with a mean of 0. The smaller the distance between θ*_*k*_* and δ*_*k*_* is, the higher the expected value of *X*_*k*_ obtained in the function. This model is expected to have the same constraints as the LIM. Furthermore, the parameter estimation of the MCMC algorithm can be adopted. In future research, the parameter recovery of this new model will be evaluated using a simulation approach similar to that reported in this study.

## Data Availability Statement

The raw data supporting the conclusions of this article will be made available by the authors, without undue reservation.

## Ethics Statement

The studies involving human participants were reviewed and approved by the Human Research Ethics Committee at the Education University of Hong Kong. Written informed consent to participate in this study was provided by the participants, and where necessary, the participants’ legal guardian/next of kin.

## Author Contributions

C-WC contributed for creating ideas, data collection, data analysis, and writing. W-CW contributed to the discussion of ideas and revision of the manuscript. MM and RS contributed to the revision of the manuscript. All authors contributed to the article and approved the submitted version.

## Conflict of Interest

The authors declare that the research was conducted in the absence of any commercial or financial relationships that could be construed as a potential conflict of interest.

## Publisher’s Note

All claims expressed in this article are solely those of the authors and do not necessarily represent those of their affiliated organizations, or those of the publisher, the editors and the reviewers. Any product that may be evaluated in this article, or claim that may be made by its manufacturer, is not guaranteed or endorsed by the publisher.

## Footnotes

1
www.questionpro.com
