# Supplementary material for: A Lognormal Ipsative Model for Multidimensional Compositional Items
Source: Front Psychol. 2021 Oct 12;12:573252. doi: 10.3389/fpsyg.2021.573252 (PMC8545823; doi:10.3389/fpsyg.2021.573252)
Supplement: Supplementary file 1 [file Data_Sheet_1.docx]

# Appendix A. Specific Objectivity for Compositional Data

## Specific Objectivity

Although Brown (2016) developed a Thurstonian model for compositional items, it is not sufficient for the good measurement property of specific objectivity. In this appendix, the importance of specific objectivity and the procedure for proving specific objectivity are described.

Rasch (1977) defined specific objectivity as a requirement of a measurement model to produce measures that are sample-free for items and test-free for respondents. Sample-free measurement means that the item difficulty estimates should remain consistent in any statistically possible persons who are measured. Similarly, test-free measurement means that the ability estimates of persons should be consistent in any statistically possible items that are used to measure the persons. Specific objectivity enables the measurement to be used for objective comparison between measures.

Essential to these comparisons is the ratio of the probability of right-to-wrong answers (Wright & Linacre, 1987), which is referred to as the persons’ odds to the item. The test-free property is achieved if, and only if, the ratio between any two persons’ odds to the identical item is the function that includes only the two persons’ abilities (and not the difficulty of the item). The sample-free property is achieved if, and only if, the ratio of one person’s odds between any two items is the function that includes only the two item difficulties (and not the ability of the person). Measurement models that satisfy specific objectivity must have both test-free and sample-free properties. Therefore, if either the test-free or the sample-free property is violated, the model will not have the property of specific objectivity. For example, the Rasch model has the property of specific objectivity. Suppose person *n* and person *m* take an identical item *i*. The log odds of person *n* to item *i* and the log odds of person *m* to the same item *i* can be written as

$\log(P_{ni}/(1-P_{ni}))=\text{θ}_{n}-\text{δ}_{i}$ and $\log(P_{mi}/(1-P_{mi}))=\text{θ}_{m}-\text{δ}_{i}$, respectively, (A1)

where *P_ni_* is the probability of person *n* correctly answering item *i*, *θ_n_* is person *n*’s ability, and *δ_i_* is the difficulty of item *i*. *P_mi_* is the probability of person *m* correctly answering item *i*, and *θ_m_* is person *m*’s ability. To demonstrate the test-free property in the Rasch model, I calculate the ratio of person *n*’s odds to person *m*’s odds.

$\log\left[\frac{P_{ni}/(1-P_{ni})}{P_{mi}/(1-P_{mi})}\right]=\text{θ}_{n}-\text{δ}_{i}-(\text{θ}_{m}-\text{δ}_{i})=\text{θ}_{n}-\text{θ}_{m}$. (A2)

The log odds ratio in Equation A2 represents the comparison between persons *m* and *n*. It involves only the two persons’ abilities and is independent of the item difficulty. In the same way, suppose person *n* takes two different items, *i* and *j*. The sample-free property can be demonstrated by observing the ratio of person *n*’s odds between items *i* and *j*.

$\log\left[\frac{P_{ni}/(1-P_{ni})}{P_{nj}/(1-P_{nj})}\right]=\text{θ}_{n}-\text{δ}_{i}-(\text{θ}_{n}-\text{δ}_{j})=-\text{δ}_{i}+\text{δ}_{j}$. (A3)

The log odds ratio involves only the two item difficulties and is independent of the person’s ability. Equations A2 and A3 illustrate that the Rasch model can claim to have the property of specific objectivity.

However, the two-parameter logistic model (2PLM) or the three-parameter logistic model (3PLM) does not have the property of specific objectivity. Let us take 2PLM as an example. The log odd of person *n* and person *m* answering an identical item *i* is

$\log(P_{ni}/(1-P_{ni}))=a_{i}(\text{θ}_{n}-\text{δ}_{i})$ and $\log(P_{mi}/(1-P_{mi}))=a_{i}(\text{θ}_{m}-\text{δ}_{i})$, (A4)

where *a_i_* is the slope parameter of item *i*. The log ratio of the two persons’ odds can be written as

$\log\left[\frac{P_{ni}/(1-P_{ni})}{P_{mi}/(1-P_{mi})}\right]=a_{i}(\text{θ}_{n}-\text{δ}_{i})-a_{i}(\text{θ}_{m}-\text{δ}_{i})=a_{i}(\text{θ}_{n}-\text{θ}_{m})$. (A5)

Clearly, the comparison between two persons depends on item slope *a_i_*. The meaning of the difference between *θ_n_* and *θ_m_* (i.e., *θ_n_* − *θ_m_*) is adjusted by the tests they took. The odds ratio of the two persons cannot be explained only by the two persons’ abilities. Therefore, 2PLM does not have the property of specific objectivity.

Nonetheless, many researchers have made comparisons using 2PLM or 3PLM. Specific objectivity may be an obscure property that is intended to be ignored, especially when using 2PLM or 3PLM, which can obtain a better model–data fit. Researchers should choose among the Rasch model, 2PLM, and 3PLM according to the study objective. When using the Rasch model, emphasis is on the meaningful comparison in the measurements and the validation of the test; when using the 2PLM or 3PLM model, emphasis is on establishing a good model–data fit. Researchers may choose to use relevant measurement models for the different purposes of their studies.

## Specific Objectivity for Compositional Data

The demonstration of specific objectivity for the Rasch model in Equations A2 and A3 uses the log ratio of odds to represent the comparison of the probabilities to answer correctly between persons (test-free) or between items (sample-free). The statistics *odds* are used because the outcome variable in the Rasch model consisted of dichotomous data (either a correct answer or an incorrect answer), and the odds effectively expresses the situation for dichotomous data. By contrast, compositional items yield continuous and ipsative response data (i.e., compositional response data). This section aims to show how specific objectivity is achieved for the compositional response data.

Here, I review the theorem of specific objectivity. Rasch (1977) gave the main theorem of specific objectivity. The transformation from a parametric reaction function into a purely additive relation is a necessary and sufficient condition for specific objectivity comparability. That is, *x*’=*b*’+*d*’, where *x*’ is the transformed outcome variable, *b*’ is the Object, and *d*’ is the Agent. The Object and the Agent can be defined as person and item, respectively, in the scenario of testing.

$x'_{mi}-x'_{ni}=b'_{m}-b'_{n}$ and $x'_{ni}-x'_{nj}=d'_{i}-d'_{j}$ (A6)

Equation A6 is the main theorem. In physics, the general gas function has specific objectivity (Rasch, 1977). It expresses the gas pressure *GP* as the relationship among the volume *V*, temperature *T*, and amount of gas *N*. The equation is originally written as *GP*=(*R*/*V*)×*N*×*T*. As it is multiplicative, a logarithm transformation makes it additive:

log(*GP*) = log(*R*/*V*) + log(*N*) + log(*T*), (A7)

where *R* is a universal constant. When the two situations *g*1 and *g*2 are different only in temperature, the comparison of the log gas pressure log(*GP*) is based on the difference between the log of the two temperatures:

log(*GP_g_*_1_) – log(*GP_g_*_2_) = log(*T_g_*_1_) – log(*T_g_*_2_). (A8)

In the testing scenario with dichotomous response data, the outcome variable is binary (correct answer or incorrect answer). By using the logit link function (log odds), person ability and item difficulty can be linear additive, as shown in Equation A1.

The Rasch model has been demonstrated to have specific objectivity (Rasch, 1977). Equation A2 and A3 represent the test-free and sample-free of the Rasch model.

In the test with compositional response data, Aitchison (1982) proposed the additive log ratio transformation to make the compositional data follow the normal distribution, that is, the logarithm of the ratio of the two elements in different dimensions (i.e., dimension *k* and dimension *D*):

$\log\left[\frac{X_{k}}{X_{D}}\right]$, (A9)

where *X_k_* and *X_D_* can be any pair of elements in the compositional data. In the parametric modeling, the additive log ratio transformation is used for the estimated value of the elements. That is,

. (A10)

Therefore, for compositional data, specific objectivity is held when the comparison of the additive log ratio transformation between persons is based only on the person parameters

, (A11)

where $\hat{X}_{ni(k)}$ and $\hat{X}_{ni(D)}$ are the point-estimated value of person *n*’s response to item *i* in dimension *k* and dimension *D*, respectively; $\hat{X}_{mi(k)}$ and $\hat{X}_{mi(D)}$ are the point-estimated value of person *m*’s response to item *i* in dimension *k* and dimension *D*, respectively; and θ*_n_*(*_k_*), θ*_n_*(*_D_*), θ*_m_*(*_k_*), and θ*_m_*(*_D_*) are person *n*’s latent trait in dimension *d*, person *n*’s latent trait in dimension *D*, person *m*’s latent trait in dimension *d*, and person *m*’s latent trait in dimension *D*, respectively. Clearly, Equation A11 shows that the difference between the two persons’ additive log ratio is the function of the two persons’ latent trait θ only. Therefore, Equation A11 is the requirement for test-free in compositional tests. Similarly, the comparison between items is based only on the statement parameters.

, (A12)

where $\hat{X}_{nj(k)}$ and $\hat{X}_{nj(D)}$ are the point-estimated value of person *n*’s response to item *j* in dimension *k* and dimension *D*, respectively, and δ*_i_*(*_k_*), δ*_i_*(*_D_*), δ*_j_*(*_k_*), and δ*_j_*(*_D_*) are the parameters of statement *i* in dimension *k*, statement *i* in dimension *D*, statement *j* in dimension *k*, and statement *j* in dimension *D*, respectively. Equation A12 is the requirement for sample-free in compositional tests. In conclusion, the demonstration of specific objectivity in compositional tests does not use the difference between logistic probabilities (logit link function) but instead used the difference between the log ratios of the expected values (additive log ratio transformation). The measurement model for compositional items can have the property of specific objectivity if the model satisfies Equations A11 (test-free) and A12 (sample-free).

Although the additive log ratio transformation involves only two elements, in the case of compositional tests with three or more dimensions, Equations A11 and A12 are still the requirement for specific objectivity because *any* pair of elements having the property indicates the whole combination of elements having the property.

**Reference**

Aitchison, J. (1982). The statistical analysis of compositional data. *Journal of the Royal Statistical Society. Series B (Methodological), 44*(2), 139–177.

Brown, A. (2016). Thurstonian scaling of compositional questionnaire data. *Multivariate Behavioral Research, 51*(2-3), 345–356.

Rasch, G. (1977). On specific objectivity. *Danish Yearbook of Philosophy, 14*, 58–94.

Wright, B. D., & Linacre, J. M. (1987). Dichotomous Rasch model derived from specific objectivity. *Rasch Measurement Transactions, 1*(1), 5–6.
