# Supplementary material for: A Lognormal Ipsative Model for Multidimensional Compositional Items
Source: Front Psychol. 2021 Oct 12;12:573252. doi: 10.3389/fpsyg.2021.573252 (PMC8545823; doi:10.3389/fpsyg.2021.573252)
Supplement: Supplementary file 2 [file Data_Sheet_2.PDF]

Appendix B. Schwartzs Value Survey – Constant Sum items

The purpose of this questionnaire is to establish the data base of compositional item responses. There is no personal identification in your response, and your contribution will be confidential. In the demographic part, please tick ☑ in the item corresponding you.

【PART I.】Demographic

1. Gender： ☐Male ☐Female
2. Age： ________________years old
3. Education level： ☐Elementary ☐High School ☐Bachelor ☐Masters or Doctorate
4. Religion： ☐Christianity/Catholic ☐Islam ☐Hinduism ☐Chinese traditional religion ☐Buddhism ☐Irreligious ☐Others : ________________________

【PART II.】Schwartzs Vaule Scale

In the following items, please allocate 100 points on how important the description of the values in your life. Give a higher number of points to the statement that is more important to you. The summation of the scores to the four statements in each item should be 100 points.

Please give the points according to the importance of the value to you. The sum of the four scores should be constant to 100.

Example:

Value	Point
(A) To try hard to avoid getting sick. Staying healthy.	25
(B) Everyone to be treated justly, even people I don't know. Protect the weak in society.	40
(C) To have surprises. To have an exciting life.	15
(D) To be ambitious. To be in charge and tell others what to do	20
Sum	100 (Fixed)

Please start to respond in the following items:

Question 1

Value	Point
(A) To help the people around me. To care for other people.	
(B) Always to behave properly. Avoid doing anything people would say is wrong.	
(C) To be rich. To have a lot of money and expensive things.	
(D) Enjoying life's pleasures. I like to 'spoil' myself.	
Sum	100 (Fixed)

Question 2

Value		Point
(A)	People should care for nature. Looking after the environment is important.	
(B)	It is best to do things in traditional ways. To follow the customs or religions we have learned.	
(C)	To show my own abilities. People to admire what I do.	
(D)	To do lots of different things in life. I always looks for new things to try.	
	Sum	100 (Fixed)

Question 3

Value		Point
(A)	Forgiving people who might have wronged me. We should see what is good in them and not to hold a grudge.	
(B)	To live in secure surroundings. Avoids anything that might endanger my safety.	
(C)	To be the one who makes the decisions, and to be the leader.	
(D)	To be interested in things, and to be curious and to try to understand all sorts of things.	
	Sum	100 (Fixed)

Question 4

Value		Point
(A)	To listen to people who are different from me. Even when I disagree with them, I still want to understand them.	
(B)	To be polite to other people all the time. Never to disturb or irritate others.	
(C)	Getting ahead in life, and to do better than others.	
(D)	To be independent, and to rely on myself.	
	Sum	100 (Fixed)

Question 5

Value		Point
(A)	To help the people around me. To care for other people.	
(B)	It is best to do things in traditional ways. To follow the customs or religions we have learned.	
(C)	To be the one who makes the decisions, and to be the leader.	
(D)	To be independent, and to rely on myself.	
	Sum	100 (Fixed)

Question 6

	Value	Point
(A)	People should care for nature. Looking after the environment is important.	
(B)	To live in secure surroundings. Avoids anything that might endanger my safety.	
(C)	Getting ahead in life, and to do better than others.	
(D)	Enjoying life's pleasures. I like to 'spoil' myself.	
	Sum	100 (Fixed)

Question 7

	Value	Point
(A)	Forgiving people who might have wronged me. We should see what is good in them and not to hold a grudge.	
(B)	To be polite to other people all the time. Never to disturb or irritate others.	
(C)	To be rich. To have a lot of money and expensive things.	
(D)	To do lots of different things in life. I always look for new things to try.	
	Sum	100 (Fixed)

Question 8

	Value	Point
(A)	To listen to people who are different from me. Even when I disagree with them, I still want to understand them.	
(B)	Always to behave properly. Avoid doing anything people would say is wrong.	
(C)	To show my own abilities. People to admire what I do.	
(D)	To be interested in things, and to be curious and to try to understand all sorts of things.	
	Sum	100 (Fixed)

Question 9

	Value	Point
(A)	To be loyal to my friends. I want to devote myself to people close to me.	
(B)	To be obedient. I should always show respect to my parents and to older people.	
(C)	To be in charge in team works	
(D)	I seek every chance I can to have fun. To do things that give me pleasure.	
	Sum	100 (Fixed)

Question 10

Value		Point
(A)	All the worlds' people should live in harmony. Promoting peace among all groups in the world is important to me.	
(B)	Not to ask for more than what you have. People should be satisfied with what they have.	
(C)	Tell others what to do. I want people to do what I say.	
(D)	I really want to enjoy life. Having a good time is very important to me.	
	Sum	100 (Fixed)

Question 11

Value		Point
(A)	Everyone should be treated justly, even people I don't know. It is important to me to protect the weak in society.	
(B)	To be humble and modest. I try not to draw attention to myself.	
(C)	Being very successful is important to me. I like to impress other people.	
(D)	I like to take risks. I am always looking for adventures.	
	Sum	100 (Fixed)

Question 12

Value		Point
(A)	It is important to me to adapt to nature and to fit into it. People should not change nature.	
(B)	Things should be organized and clean. I don't want things to be a mess.	
(C)	To be ambitious. I want to show how capable I am.	
(D)	Thinking up new ideas and being creative is important to me. I like to do things in my own original way.	
	Sum	100 (Fixed)

Question 13

Value		Point
(A)	To be loyal to my friends. I want to devote myself to people close to me.	
(B)	Not to ask for more than what you have. People should be satisfied with what they have.	
(C)	Being very successful is important to me. I like to impress other people.	
(D)	Thinking up new ideas and being creative is important to me. I like to do things in my own original way.	
	Sum	100 (Fixed)

Question 14

	Value	Point
(A)	All the worlds' people should live in harmony. Promoting peace among all groups in the world is important to me.	
(B)	To be humble and modest. I try not to draw attention to myself.	
(C)	To be ambitious. I want to show how capable I am.	
(D)	I seek every chance I can to have fun. To do things that give me pleasure.	
	Sum	100 (Fixed)

Question 15

	Value	Point
(A)	Everyone should be treated justly, even people I don't know. It is important to me to protect the weak in society.	
(B)	Things should be organized and clean. I don't want things to be a mess.	
(C)	To be in charge in team works	
(D)	I really want to enjoy life. Having a good time is very important to me.	
	Sum	100 (Fixed)

Question 16

	Value	Point
(A)	It is important to me to adapt to nature and to fit into it. People should not change nature.	
(B)	To be obedient. I should always show respect to my parents and to older people.	
(C)	Tell others what to do. I want people to do what I say.	
(D)	I like to take risks. I am always looking for adventures.	
	Sum	100 (Fixed)

Question 17

	Value	Point
(A)	All the worlds' people should live in harmony. Promoting peace among all groups in the world is important to me.	
(B)	To be obedient. I should always show respect to my parents and to older people.	
(C)	Getting ahead in life, and to do better than others.	
(D)	To be interested in things, and to be curious and to try to understand all sorts of things.	
	Sum	100 (Fixed)

Question 18

Value		Point
(A)	Forgiving people who might have wronged me. We should see what is good in them and not to hold a grudge.	
(B)	Not to ask for more than what you have. People should be satisfied with what they have.	
(C)	To be in charge in team works	
(D)	To be independent, and to rely on myself.	
	Sum	100 (Fixed)

Question 19

Value		Point
(A)	To listen to people who are different from me. Even when I disagree with them, I still want to understand them.	
(B)	To live in secure surroundings. Avoids anything that might endanger my safety.	
(C)	Tell others what to do. I want people to do what I say.	
(D)	I seek every chance I can to have fun. To do things that give me pleasure.	
	Sum	100 (Fixed)

Question 20

Value		Point
(A)	To be loyal to my friends. I want to devote myself to people close to me.	
(B)	To be polite to other people all the time. Never to disturb or irritate others.	
(C)	To be the one who makes the decisions, and to be the leader.	
(D)	I really want to enjoy life. Having a good time is very important to me.	
	Sum	100 (Fixed)

Question 21

Value		Point
(A)	People should care for nature. Looking after the environment is important.	
(B)	Always to behave properly. Avoid doing anything people would say is wrong.	
(C)	To be ambitious. I want to show how capable I am.	
(D)	I like to take risks. I am always looking for adventures.	
	Sum	100 (Fixed)

Question 22

	Value	Point
(A)	Everyone should be treated justly, even people I don't know. It is important to me to protect the weak in society.	
(B)	It is best to do things in traditional ways. To follow the customs or religions we have learned.	
(C)	To be rich. To have a lot of money and expensive things.	
(D)	Thinking up new ideas and being creative is important to me. I like to do things in my own original way.	
	Sum	100 (Fixed)

Question 23

	Value	Point
(A)	It is important to me to adapt to nature and to fit into it. People should not change nature.	
(B)	To be humble and modest. I try not to draw attention to myself.	
(C)	To show my own abilities. People to admire what I do.	
(D)	Enjoying life's pleasures. I like to 'spoil' myself.	
	Sum	100 (Fixed)

Question 24

	Value	Point
(A)	To help the people around me. To care for other people.	
(B)	Things should be organized and clean. I don't want things to be a mess.	
(C)	Being very successful is important to me. I like to impress other people.	
(D)	To do lots of different things in life. I always looks for new things to try.	
	Sum	100 (Fixed)

Question 25

	Value	Point
(A)	To help the people around me. To care for other people.	
(B)	To live in secure surroundings. Avoids anything that might endanger my safety.	
(C)	To be in charge in team works	
(D)	I like to take risks. I am always looking for adventures.	
	Sum	100 (Fixed)

Question 26

Value	Point
(A) Forgiving people who might have wronged me. We should see what is good in them and not to hold a grudge.	
(B) To be obedient. I should always show respect to my parents and to older people.	
(C) Being very successful is important to me. I like to impress other people.	
(D) Enjoying life's pleasures. I like to 'spoil' myself.	
Sum	100 (Fixed)

Question 27

Value	Point
(A) To be loyal to my friends. I want to devote myself to people close to me.	
(B) To be humble and modest. I try not to draw attention to myself.	
(C) To be rich. To have a lot of money and expensive things.	
(D) To be interested in things, and to be curious and to try to understand all sorts of things.	
Sum	100 (Fixed)

Question 28

Value	Point
(A) Everyone should be treated justly, even people I don't know. It is important to me to protect the weak in society.	
(B) Always to behave properly. Avoid doing anything people would say is wrong.	
(C) To be the one who makes the decisions, and to be the leader.	
(D) I seek every chance I can to have fun. To do things that give me pleasure.	
Sum	100 (Fixed)

Question 29

Value	Point
(A) People should care for nature. Looking after the environment is important.	
(B) To be polite to other people all the time. Never to disturb or irritate others.	
(C) Tell others what to do. I want people to do what I say.	
(D) Thinking up new ideas and being creative is important to me. I like to do things in my own original way.	
Sum	100 (Fixed)

Question 30

Value		Point
(A)	To listen to people who are different from me. Even when I disagree with them, I still want to understand them.	
(B)	Not to ask for more than what you have. People should be satisfied with what they have.	
(C)	To be ambitious. I want to show how capable I am.	
(D)	To do lots of different things in life. I always looks for new things to try.	
	Sum	100 (Fixed)

Question 31

Value		Point
(A)	All the worlds' people should live in harmony. Promoting peace among all groups in the world is important to me.	
(B)	Things should be organized and clean. I don't want things to be a mess.	
(C)	To show my own abilities. People to admire what I do.	
(D)	To be independent, and to rely on myself.	
	Sum	100 (Fixed)

Question 32

Value		Point
(A)	It is important to me to adapt to nature and to fit into it. People should not change nature.	
(B)	It is best to do things in traditional ways. To follow the customs or religions we have learned.	
(C)	Getting ahead in life, and to do better than others.	
(D)	I really want to enjoy life. Having a good time is very important to me.	
	Sum	100 (Fixed)

Question 33

Value		Point
(A)	To help the people around me. To care for other people.	
(B)	To be polite to other people all the time. Never to disturb or irritate others.	
(C)	Being very successful is important to me. I like to impress other people.	
(D)	To do lots of different things in life. I always look for new things to try.	
	Sum	100 (Fixed)

Question 34

	Value	Point
(A)	People should care for nature. Looking after the environment is important.	
(B)	To be obedient. I should always show respect to my parents and to older people.	
(C)	To be ambitious. I want to show how capable I am.	
(D)	To be interested in things, and to be curious and to try to understand all sorts of things.	
	Sum	100 (Fixed)

Question 35

	Value	Point
(A)	Forgiving people who might have wronged me. We should see what is good in them and not to hold a grudge.	
(B)	Not to ask for more than what you have. People should be satisfied with what they have.	
(C)	To be rich. To have a lot of money and expensive things.	
(D)	To be independent, and to rely on myself.	
	Sum	100 (Fixed)

Question 36

	Value	Point
(A)	To listen to people who are different from me. Even when I disagree with them, I still want to understand them.	
(B)	To be humble and modest. I try not to draw attention to myself.	
(C)	To show my own abilities. People to admire what I do.	
(D)	I seek every chance I can to have fun. To do things that give me pleasure.	
	Sum	100 (Fixed)

Question 37

	Value	Point
(A)	To be loyal to my friends. I want to devote myself to people close to me.	
(B)	Things should be organized and clean. I don’t want things to be a mess.	
(C)	To be the one who makes the decisions, and to be the leader.	
(D)	I really want to enjoy life. Having a good time is very important to me.	
	Sum	100 (Fixed)

Question 38

Value		Point
(A)	All the worlds' people should live in harmony. Promoting peace among all groups in the world is important to me.	
(B)	Always to behave properly. Avoid doing anything people would say is wrong.	
(C)	Getting ahead in life, and to do better than others.	
(D)	I like to take risks. I am always looking for adventures.	
	Sum	100 (Fixed)

Question 39

Value		Point
(A)	Everyone should be treated justly, even people I don't know. It is important to me to protect the weak in society.	
(B)	It is best to do things in traditional ways. To follow the customs or religions we have learned.	
(C)	To be in charge in team works	
(D)	Thinking up new ideas and being creative is important to me. I like to do things in my own original way.	
	Sum	100 (Fixed)

Question 40

Value		Point
(A)	It is important to me to adapt to nature and to fit into it. People should not change nature.	
(B)	To live in secure surroundings. Avoids anything that might endanger my safety.	
(C)	Tell others what to do. I want people to do what I say.	
(D)	Enjoying life's pleasures. I like to 'spoil' myself.	
	Sum	100 (Fixed)

Thank you for your participation!
