# Supplementary material for: A Lognormal Ipsative Model for Multidimensional Compositional Items
Source: Front Psychol. 2021 Oct 12;12:573252. doi: 10.3389/fpsyg.2021.573252 (PMC8545823; doi:10.3389/fpsyg.2021.573252)
Supplement: Supplementary file 3 [file Data_Sheet_3.PDF]

Appendix C. JAGS codes for lognormal ipsative model

```
for (j in 1:N) {
for (i in 1:I) {
for (dd in 1:Dn-1){

     TotalU[j, i, dd] <-    (theta[j, dd]      + Delta[BlkStmt[i, dd]]    ) - (theta[j, Dn]      +
Delta[BlkStmt[i, Dn]]    ) ;
    resp[j, (Dn-1)*(i-1)+dd] ~ dlnorm(TotalU[j, i, dd], tau);
}}}
tau ~ dgamma(1,1);

# Prior and hyper-prior for person parameters: Multivariate normal distribution
for (j in 1:N) {
    theta[j, Dn] <- -sum(theta[j, 1:Dn-1]);
    theta[j,1:Dn-1] ~ dmnorm(mu[1:Dn-1], CovR[1:Dn-1,1:Dn-1]) ;
}

# Mean
for (dd in 1:Dn-1){
mu[dd] <- dnorm(0, 1)
}

# Var-Cov matrices
CovR[1:Dn-1,1:Dn-1] ~ dwish(Omega[1:Dn-1,1:Dn-1], 3)
IR[1:Dn-1,1:Dn-1] <- inverse(CovR[1:Dn-1,1:Dn-1])

corr12 <- IR[1,2]/(sqrt(IR[1,1]*IR[2,2]))
corr13 <- IR[1,3]/(sqrt(IR[1,1]*IR[3,3]))
corr23 <- IR[2,3]/(sqrt(IR[2,2]*IR[3,3]))

# Prior for item location parameters
for (dd in 1:Dn){
    Delta[DIn*dd] <- -sum(Delta[(DIn*(dd-1)+1):(DIn*dd-1)]);
}

    for (dd in 1:Dn){
        for (dId in 1:DIn-1) {
        Delta[(dd-1)*DIn+dId] ~ dnorm(0,1);
    }}
```
